# The adaptive functional piecewise ordered weighted averaging method and its application to pollutant concentration analysis

**DOI:** 10.1371/journal.pone.0342192

**Published:** 2026-02-13

**Authors:** Yang Li, Xiaoxue Hu, Maozai Tian

**Affiliations:** 1 School of Statistics and Data Science, Xinjiang University of Finance and Economics, Urumqi, Xinjiang, China; 2 Center for Applied Statistics, School of Statistics, Renmin University of China, Beijing, China; Istinye University: Istinye Universitesi, TÜRKIYE

## Abstract

The evolving patterns of pollutant concentrations and their rigorous assessment are critical issues in contemporary environmental research and policy-making, with important practical implications for air quality management and regional pollution control. To better support such decisions, scientifically sound multi-criteria ranking methods have become a key research focus. In this paper, we propose a novel adaptive functional piecewise ordered weighted averaging (FP-OWA) method for ranking complex functional data. The method extends the existing functional piecewise ranking–weighting framework by integrating data smoothing, depth-based centrality measures, and rank-based aggregation. We systematically compare the performance of FP-OWA with several existing functional data ranking methods using Monte Carlo simulations. The results show that FP-OWA substantially improves ranking consistency and stability when the data are contaminated by white noise. We further apply FP-OWA to rank the daily average *PM*2.5 and *O*_3_ concentrations in 13 cities in the Beijing–Tianjin–Hebei region in 2023, accurately revealing the spatiotemporal differentiation patterns of regional pollution. These findings provide a solid technical basis for local governments to design pollution control strategies and improve air quality. Future research will focus on extending FP-OWA to highly nonlinear and complex functional data, further enhancing its computational efficiency to meet big-data processing requirements, and exploring additional application scenarios.

## 1 Introduction

Air pollution is a major environmental problem that affects human health, ecosystems, and the sustainable development of economies and societies worldwide. As a key economic growth center and urban agglomeration in northern China, the Beijing–Tianjin–Hebei region has attracted considerable attention due to its high population density, complex pollution sources, and pronounced regional transport characteristics. The complexity of its air pollution is reflected in the overlapping of multiple emission sources, the heterogeneity of spatiotemporal distribution, and the influence of inter-regional transport. First, the Beijing–Tianjin–Hebei region is characterized by diverse pollution sources, including industrial emissions, traffic exhaust, coal combustion, dust, and volatile organic compounds, with the contribution of each source varying substantially across cities [1]. Moreover, accelerated regional integration has led to traffic congestion, surging energy consumption, and concentrated pollutant emissions, further highlighting the difficulty of managing the superposition of multiple pollution sources [2]. Second, air pollution in the Beijing–Tianjin–Hebei region exhibits distinctive seasonal and spatial patterns. Higher concentrations of *PM*2.5 and *NO*_*x*_ are observed in winter due to coal-fired heating and unfavorable meteorological conditions, while in summer, high temperatures and strong solar radiation promote photochemical reactions, exacerbating *O*_3_ pollution [3]. Spatially, annual average *PM*2.5 concentrations in cities in southern Hebei Province (such as Shijiazhuang, Xingtai, and Handan) are significantly higher than in Beijing and Tianjin because their industrial structures remain heavily dominated by secondary industry, resulting in a pronounced “low in the north, high in the south” gradient [4,5]. Furthermore, as part of the North China Plain, the topography and meteorological conditions of the Beijing–Tianjin–Hebei region intensify cross-regional transport of pollutants [6,7].

However, when confronted with complex and diverse air pollution problems, traditional assessment methods such as principal component analysis (PCA) and the analytic hierarchy process (AHP) have limited ability to capture interaction effects and spatiotemporal variation among pollutants in dynamic, high-dimensional settings. In contrast, functional data analysis (FDA) offers a new framework for handling complex environmental data by transforming discrete observations into continuous functions [8]. FDA began to develop in the early 1990s. Ramsay and Silverman [9] systematically presented key methods such as smoothing techniques, functional principal component analysis (FPCA), and functional linear models, and established the theoretical foundation of the field. Their work details the use of spline functions and Fourier bases to smooth observations, effectively suppressing noise while preserving the continuity of the data. Ferraty and Vieu [10] made important contributions to nonparametric methods, opening new avenues for functional data classification and regression. Horváth and Kokoszka [11] focused on statistical inference for functional data, including hypothesis testing and the construction of confidence intervals.

Data ranking problems are a key challenge in FDA applications. Existing functional data ranking methods can be broadly categorized into three classes: embedding-based, distance-based, and depth-based approaches. Embedding-based ranking methods typically map high-dimensional functional data into a low-dimensional space to simplify computation and the ranking procedure. A classical example is the Fourier transform, which extracts frequency features by transforming data from the time or spatial domain to the frequency domain, thereby enabling effective ranking of functional data with periodic variation [12]. PCA, as another embedding technique, performs linear dimensionality reduction by extracting principal component directions. This approach reduces dimensionality while preserving the main information in the data, thus improving ranking efficiency. However, its inherent linearity may limit its effectiveness when the underlying data structure is strongly nonlinear [13].

Distance-based metrics play an important role in functional data ranking. The core idea is to obtain a ranking by quantifying the dissimilarity between functions. Commonly used metrics include the *L*_2_ norm, Dynamic Time Warping (DTW), and the Fréchet distance. The *L*_2_ norm, as a basic measure, computes the integral of the squared difference between functions. Although it is computationally simple and easy to interpret, it cannot accommodate time shifts and may therefore fail to capture key curve features when analyzing nonlinear functional data. By optimizing the alignment path between curves, DTW allows elastic scaling along the time axis and effectively addresses phase differences in time series [14]. It has been widely applied in fields such as speech recognition and data mining [15]. The Fréchet distance evaluates curve similarity from a geometric perspective, taking into account both spatial location and continuity, which makes it particularly suitable for analyzing the overall structural characteristics of functions [16]. Other metrics, such as the Hausdorff distance and Wasserstein distance, are also useful in specific scenarios, but they generally exhibit limited robustness to noise. Wang et al. [17] showed that although DTW is effective for nonlinear time series, noise contamination can easily cause instability in the resulting rankings.

To overcome the limitations of traditional distance-based metrics, ranking methods based on statistical depth have been developed. These approaches originate from multivariate statistical analysis, where Tukey’s [18] pioneering work on half-space depth initiated systematic research on depth notions, followed by variants such as simplex depth and projection depth. Considering the infinite-dimensional nature of functional data, López-Pintado and Romo [19] proposed the concept of band depth (BD). In this framework, the depth of a curve is determined by evaluating its “centrality” within the sample: specifically, by computing the probability that the target curve lies within the band formed by other curves. A larger depth value indicates that the curve is closer to the core of the data distribution and therefore more representative of the overall pattern. This property allows BD to maintain stable ranking performance even in the presence of noise or outliers [20], and it has been successfully applied to dynamic functional data in areas such as environmental science and financial analysis. For example, in air quality monitoring, identifying the most representative pollutant concentration trajectories provides a sound scientific basis for environmental policy-making [21]. The subsequent Modified Band Depth (MBD) further improves ranking accuracy by incorporating amplitude information. New depth notions, such as h-mode depth [22] and random Tukey depth [23], have also introduced additional perspectives to this research field. Although depth-based methods offer clear advantages in terms of robustness to noise and outliers, the choice of depth measures and the tuning of associated parameters still require further investigation. Sun and Genton [24] applied BD to construct functional boxplots, which not only facilitate data visualization but also provide an effective tool for anomaly detection, thereby highlighting the practical value of depth-based methods.

With its core advantage of modeling discrete observations as continuous functions, functional data analysis (FDA) has become a key tool for addressing complex spatiotemporal dynamics in environmental and climate research. In recent years, numerous innovative studies have emerged, including applications to fine particulate matter assessment and analyses of the relationship between carbon emissions and economic activity. King et al. [25], for example, developed an ST-FDA model to investigate the spatiotemporal variation of fine particulate matter components in the United States. Their approach treats annual concentration curves as functional time series, decomposes the covariance function via FPCA, and uses Kriging to predict concentrations at unobserved locations. Using IMPROVE and CSN network data from 2003 to 2015, they identified key patterns such as higher nitrate levels in urban than in rural areas, seasonal peaks in colder months, and an overall downward trend over time. In the climate–economy domain, Elayouty and Abou-Ali [26] applied FDA methods to assess the dynamic effects of electricity consumption and economic growth on *CO*_2_ emissions from 1975 to 2014. They employed FPCA to extract the main modes of variation in emission trajectories, combined with functional linear regression to quantify the time-varying relationships among electricity consumption, economic growth, and *CO*_2_ emissions. An EM-based clustering procedure was then used to group countries into five distinct emission pathways, providing a refined empirical basis for climate policy design. Although Notter [27] focused on life cycle impact assessment (LCIA) of particulate matter and did not explicitly adopt the FDA framework, the continuous modeling of physicochemical properties—such as particle size in the 1nm−10μm range and 34 chemical components—closely parallels FDA ideas. Through four modules (Fate, Exposure, Effect, and Damage), the study transformed particulate matter emissions into a continuous health-damage function, quantified the differentiated toxic effects of particle size and solubility, and addressed limitations in traditional LCIA methods. This provides valuable methodological guidance for future FDA applications to the continuous dynamic assessment of particulate matter toxicity. Akopov et al.’s [28] ecological–economic modeling of Armenian enterprises, in which indicators such as industrial output and emissions are treated as time-dependent variables, also offers inspiration for extending FDA to ecological economics. In future work, it would be promising to represent the dynamic relationship between enterprise emissions and output as functional trajectories using FDA, and to combine this with functional deep ranking and related methods to identify optimal transformation paths that balance ecological and economic benefits, thereby further enriching cross-disciplinary applications of FDA.

This paper tackles the problem of functional data ranking by proposing an innovative method that integrates the strengths of several existing techniques, with a particular focus on improving the stability and adaptability of ranking procedures. The main contributions of this study can be summarized in three aspects. First, leveraging the continuous nature of functional data, spline smoothing is applied to preprocess the original observations, effectively filtering out high-frequency noise. Second, a density-based DBSCAN clustering algorithm is introduced to partition the functional data into several sub-intervals, within which a rank-based strategy is implemented to substantially reduce the influence of abnormal observations on the final ranking. Third, the Modified Band Depth (MBD) is incorporated to quantify the centrality of each curve and construct segment-level weights, thereby enhancing the robustness of the ranking with respect to outliers.

The remainder of this paper is organized as follows. Sect 2 reviews existing functional ranking methods and presents the proposed FP-OWA approach. Sect 3 discusses the asymptotic properties of the proposed estimator. Sect 4 reports extensive numerical experiments that evaluate the performance of the method under different scenarios. Sect 5 applies FP-OWA to rank the 2023 daily average *PM*_2.5_ and *O*_3_ concentrations in 13 cities in the Beijing–Tianjin–Hebei region, providing a solid technical basis for formulating regional pollution control strategies and improving air quality. Finally, Sect 6 concludes the paper and outlines potential directions for future research.

## 2 Introduction to ranking methods

### 2.1 Existing functional ranking methods

FPCA is a dimensionality reduction technique for functional data that captures the main modes of variation over a continuous domain, thereby reducing dimensionality while preserving essential features. First, to reflect the continuous nature of the data, discrete observations are smoothed—typically using spline or kernel smoothing—to transform the observed points into continuous curves *X*_*i*_(*t*). Next, the sample covariance function *C*(*s*,*t*) is estimated and subjected to eigen-decomposition to obtain eigenvalues $\lambda_{k}$ and eigenfunctions $\phi_{\dot{\kappa }}(t)$. On the basis of $\lambda_{k}$, the cumulative proportion of variance γ(K) is calculated to determine the number of principal components. The principal component scores $\xi_{ik}$ for each sample curve are then computed using the eigenfunctions $\phi_{\dot{\kappa }}(t)$. Finally, the samples are ranked according to these scores. In most applications, the first principal component score $\xi_{i1}$, is used as the primary ranking criterion. When multiple principal components are used, a composite score $Score_{i}=\sum_{k=1}^{k}w_{k}\xi_{ik}$, is constructed, where $w_{k}=\frac{\lambda_{k}}{\sum_{j=1}^{K}\lambda_{j}}$. FPCA can eliminate redundant dimensions and effectively capture dynamic variation patterns in functional data. However, it is sensitive to noise and outliers, the selection of eigenfunctions depends on accurate covariance estimation, and nonlinear dependencies cannot be directly accommodated.

WLR is a functional data analysis method for time series that transforms discrete observations into continuous curves and produces robust ranking results through segmentation and weighting [29]. Using piecewise linear interpolation. When handling missing values, if a missing value occurs at an intermediate time point, that point is skipped and the adjacent observed points are directly connected; if the missing value is located at either endpoint, multiple imputation is used to complete the data. Once the continuous function is obtained, the time series is divided into several segments. Suppose *Y*_*i*_(*t*) is partitioned into msegments over the interval $\left[t_{0},t_{m}\right]$, with segment boundaries $\left\{t_{0},t_{1},\ldots ,t_{m}\right\}$. The segment weight *w*_*j*_ is calculated according to the proportion of the length of the *j*-th segment relative to the total duration of the interval. To reduce the influence of outliers, observations within each segment are ranked. Specifically, in the *j*-th segment, let the observations of the *i*-th sample be $Y_{i}(t_{k})$ for $t_{\dot{\kappa }}\in \left[t_{j-1},t_{j}\right]$, the rank statistic *R*_*ij*_ is then computed for each sample, with average ranks assigned in the case of ties. Finally, a composite score for each sample is obtained using the segment weights *w*_*j*_ and rank values *R*_*ij*_, expressed as $Score_{i}=\sum_{j=1}^{m}w_{j}R_{ij}$. Through segmented processing, WLR partially preserves the continuity and temporal characteristics of the data, making it suitable for scenarios with relatively stable temporal dynamics. However, its ranking results can be sensitive to the choice of segment length and the number of observation points. In addition, WLR does not explicitly account for the central tendency of the data and remains relatively sensitive to outliers.

The h-mode depth, proposed by Cuevas et al. [30], is a depth measure based on nonparametric kernel density estimation. Unlike depth notions defined in terms of geometric position, h-mode depth focuses on the local probability density of curves in the function space. It can effectively identify the most densely populated regions of the distribution and is particularly suitable for capturing the central features of asymmetric or multimodal functional data. Let $X_{1}(t),\ldots ,X_{n}(t)$ be functional observations on an interval *T*. For any function *x*(*t*) its h-mode depth is defined as


$$D_{h}(x)=\frac{1}{n}\sum_{i=1}^{n}K\left(\frac{∥x-X_{i}∥}{h}\right),$$

where K(·) is a kernel function and h is a bandwidth parameter controlling the neighborhood size. A curve with a larger value of *D*_*h*_(*x*) has more neighboring sample curves in its vicinity and is therefore more representative of the overall distribution. Given the sample $X_{1}(t),\ldots ,X_{n}(t)$ and the depth measure *D*_*h*_, the ranking is obtained by ordering the sample curves in decreasing depth, that is, from the largest to the smallest value of *D*_*h*_.

Tukey depth is a classical centrality measure in multivariate statistics, but its direct computation in infinite-dimensional function spaces faces severe computational complexity. To address this issue, Cuesta-Albertos et al. [23] proposed the random Tukey depth (RTD), which uses random projections to reduce high-dimensional calculations to a sequence of one-dimensional problems, thereby substantially lowering computational cost while preserving key statistical properties. Let *x* be the function to be evaluated, and let $u_{1},\ldots ,u_{k}$ denote a set of unit projection directions drawn at random from a Sobolev space. For each direction *u*_*j*_, one computes the univariate Tukey depth of the projection $\langle x,u_{j}\rangle$. The RTD of *x* is then defined as the minimum of these univariate depths


$$RTD(x)=\min_{j=1,\ldots ,k}D_{1}\left(\langle x,u_{j}\rangle ;\{\langle X_{1},u_{j}\rangle ,\ldots ,\langle X_{n},u_{j}\rangle \}\right),$$

where *D*_1_(*y;Z*) denotes the univariate Tukey depth. RTD is affine-invariant and highly sensitive to shape outliers. Its core idea follows the minimum-depth principle: if a curve appears as an outlier along at least one projection direction, it is regarded as an outlier in the overall functional space. The ranking rule induced by RTD is consistent with that of the h-MD described above.

### 2.2 Adaptive Functional Piecewise Ordered Weighted Averaging Method (FP-OWA)

Motivated by the limitations of the existing ranking methods, we propose an adaptive functional piecewise ordered weighted averaging (FP-OWA) procedure that combines spline smoothing, measures of data centrality, and band-depth–based weighting. The goal is to enhance the robustness and adaptability of functional rankings. The definition and computation of FP-OWA are described below.

Functional observations are typically recorded at discrete time points and are often contaminated by noise and missing values. Directly performing ranking analysis on such raw discrete data may deteriorate both the accuracy and stability of the results. To alleviate these issues, we first smooth the data and impute missing values so as to obtain continuous and stable sample trajectories. Assume that each sample satisfies

$$y_{i}(t_{j})=f_{i}(t_{j})+\varepsilon_{i}(t_{j}).$$ (1)

Where, $y_{i}(t_{j})$ is the observation of the *i*-th curve at time *t*_*j*_, $f_{i}(t_{j})$ is the underlying true function value, and $\varepsilon_{i}(t_{j})$ is random noise with $E[\varepsilon_{i}(t_{j})]=0$ and $V[\varepsilon_{i}(t_{j})]=\delta^{2}$. To estimate *f*_*i*_(*t*), we expand it in a spline basis,

$$f_{i}(t)=\sum_{k=1}^{K}\beta_{\dot{\kappa }}\phi_{\dot{\kappa }}(t).$$ (2)

Here, $\phi_{\dot{\kappa }}(t)$ represents the basis functions, $\beta_{\dot{\kappa }}$ are the unknown coefficients, and K is the number of basis functions.

Minimizing the residual sum of squares alone may lead to overfitting and spurious oscillations around noisy observations. We therefore introduce a roughness penalty to balance fidelity to the data and smoothness of the estimated function. The objective function for the *i*-th curve is

$$J(c)=\sum_{j=1}^{n}{\left[y_{i}(t_{j})-\sum_{k=1}^{K}\beta_{ik}\phi_{k}(t_{j})\right]}^{2}+\lambda \int_{a}^{b}{\left[f_{i}^{\prime \prime }(t)\right]}^{2}dt,$$ (3)

where the first term measures the data-fitting error and the second term penalizes curvature. The smoothing parameter *λ* controls the trade-off between these two components. In this study, *λ* is selected by generalized cross-validation (GCV),

$$GCV(\lambda )=\frac{SSE(\lambda )}{{\left(n-df(\lambda )\right)}^{2}},$$ (4)

and the optimal value is obtained by minimizing GCV(λ). Missing observations are imputed using spline interpolation, yielding a complete smoothed trajectory ${\hat{f}}_{i}(t)$ for each curve, which facilitates the subsequent segmentation step.

The smoothed functional data typically exhibit different local behaviours over the time domain. To accurately capture these local characteristics, we segment the time axis into several subintervals according to the similarity of temporal patterns, rather than directly working with the raw observation times. Since local density changes are difficult to detect in the original time scale, we transform the segmentation problem into a clustering problem in a multidimensional feature space and employ the density-based DBSCAN algorithm to automatically identify stable time intervals.

Unlike traditional segmentation methods that rely on geometric intersections, we define similarity among time points in terms of functional features. At each time, we consider not only the function value but also its first and second derivatives. An initial feature vector is constructed as $x_{t}=\left[f(t),\vartheta_{1}f^{\prime }(t),\vartheta_{2}f^{\prime \prime }(t)\right]$. Where *f*(*t*), $f^{\prime }(t)$ and $f^{\prime \prime }(t)$ are normalized function value, first derivative, and second derivative, respectively, and $\vartheta_{1}$ and $\vartheta_{2}$ are tuning constants. To remove potential multicollinearity and extract the main modes of variation, we standardize the feature matrix and perform principal component analysis (PCA), retaining the first qprincipal components as the final *q*-dimensional feature vector *z*_*t*_. Each time point on the original axis is thus mapped to a point in feature space; if two time points are close in this space, it indicates that the values, rates of change, and curvature of all sample curves are highly consistent at these times, suggesting a locally stable regime.

DBSCAN clustering is then applied to the feature vectors *z*_*t*_. The algorithm does not require the number of clusters to be specified in advance and instead classifies points into core points, border points, and noise according to local density. It involves two key parameters: the neighborhood radius *ε*(eps) and the minimum number of points in a neighborhood, minPts. To reduce subjectivity, we use a grid-search strategy rather than ad-hoc rules:

(1) For *ε*, we adopt the k-nearest-neighbor distance elbow method, computing for each feature point the Euclidean distance to its *k*-th nearest neighbor and setting the search range for to the 10–95% quantiles of these distances.

(2) For minPts, the search range covers values from a small sample size (twice the feature dimension [31]) to relatively large, stable values.

For each parameter pair(*ε*, minPts), DBSCAN is run and the average silhouette coefficient is computed,


$$SC=\frac{1}{T}\sum_{t=1}^{T}\frac{b(t)-a(t)}{\max\left\{a(t),b(t)\right\}}.$$

Where $a(t)=\frac{1}{\left|C_{i}\right|-1}\sum_{s\in C_{i}}a\left(z_{t}-z_{s}\right),t\neq s$ is the within-cluster dissimilarity of time point *t* belonging to cluster *C*_*i*_ with $\left|C_{i}\right|$ members, $a\|z_{t}-z_{s}\|$ is the Euclidean distance in feature space, and $b(t)=\min_{C_{j}\neq C_{i}}\left\{\frac{1}{\left|C_{j}\right|}\sum_{s\in C_{j}}\bar{a}\left(z_{t}-z_{s}\right)\right\}$ is the separation from the nearest neighboring cluster *C*_*j*_. The parameter combination that maximizes *SC* is selected as optimal.

Using the optimal *ε* and minPts, we perform DBSCAN clustering on the standardized features. Each time point receives a cluster label (core, border, or noise), with noise typically labeled as 0. To preserve temporal continuity, noise labels are replaced by the labels of neighboring non-noise points. Along the time axis, a segmentation boundary is declared between two adjacent time points only if their (adjusted) cluster labels differ. The resulting breakpoints $t_{1},t_{2},\ldots ,t_{m}$ partition the entire time domain into *m* contiguous segments.

After segmentation, different segments may exhibit distinct patterns of functional variation. To quantify the representativeness of each segment, we employ the modified band depth (MBD) to measure how centrally a curve lies relative to all curves within a segment: the larger the MBD, the closer the curve is to the overall trend. For the *i*-th segment, let $f_{1},...,f_{n}$ denote the sample curves restricted to this segment. The MBD of curve *f*_*j*_(*t*) in segment *i* is

$$d_{ij}=MBD\left(f_{j};\{f_{1},...,f_{n}\}\right)\;=\frac{2}{N_{i}(N_{i}-1)}\sum_{1\leq k\leq l\leq N_{i}}\frac{1}{\left|T_{i}\right|}\int_{T_{i}}I\left\{\min\{f_{k}(t),f_{l}(t)\}\leq f_{j}(t)\leq \max\{f_{k}(t),f_{l}(t)\}\right\}dt$$ (5)

Among them, *N*_*i*_ is the number of curves in segment *i*, *T*_*i*_ is the time interval of that segment, $\left|T_{i}\right|$ is its length, and I{·} is the indicator function, which equals 1 if *f*_*j*_(*t*) lies between *f*_*k*_(*t*) and *f*_*l*_(*t*) at time *t* and 0 otherwise.

The average MBD score within segment *i* is

$${\overset{―}{d}}_{i}=\frac{1}{N_{i}}\sum_{j=1}^{N_{i}}d_{ij},$$ (6)

and the segment weights are obtained by normalizing these averages,

$$w_{i}=\frac{{\bar{d}}_{i}}{\sum_{l=1}^{n}{\overset{―}{d}}_{l}}.$$ (7)

Thus, segments whose curves are more centrally located in the sense of MBD receive larger weights in the final ranking.

Within each segment, the integral of a curve reflects its cumulative behavior over that time interval. Because raw integral values may be affected by scale and numerical range, we convert them into ranks to mitigate the influence of magnitude differences and extreme values.

For the *j*-th curve in segment *i*, in segment *i*, we define the segment-wise integral as

$$I_{ij}=\int_{a}^{b}f_{j}(t)dt.$$ (8)

Where $\left[\begin{array}{c} a,b \end{array}\right]$ is the time interval of segment *i*. Using the spline expansion of *f*_*j*_(*t*) the integral can be written as

$$I_{ij}=\sum_{k=1}^{K}c_{jk}\int_{a}^{b}\phi_{k}(t)dt.$$ (9)

After computing *I*_*ij*_ for all curves in segment *i*, we rank these values in ascending order and obtain the rank statistic *R*_*ij*_, which represents the relative position of curve *j* within that segment. Ties are handled by assigning average ranks.

Finally, to derive a global score for each curve, we aggregate the segment-wise ranks using the MBD-based weights

$$Score_{j}=\sum_{i=1}^{n}w_{i}\cdot R_{ij},$$ (10)

where a large *Score*_*j*_ indicates that curve *j* tends to occupy better positions across the segments. These overall scores induce the final FP-OWA ranking of the functional data.

### 2.3 Time complexity of ranking methods

To comprehensively assess the computational feasibility of the FP-OWA method, as well as FPCA, WLR, h-MD, and RTD methods, particularly in scenarios where the sample size *N* increases significantly or the number of time points per sample *T* grows substantially, this section discusses the asymptotic time complexity of the aforementioned methods. Suppose the dataset consists of *N* independent samples, and each sample has function data observed at *T* time points. Additionally, we assume all methods are implemented in a standard computational environment and do not account for constant factors or lower-order terms. Furthermore, for methods involving segmentation, we introduce the average number of segments *S* to quantify the impact of segmentation on overall complexity.

For the FPCA method, the computational procedure can be decomposed into several main steps. First, data smoothing is performed using spline basis functions [26], with a computational complexity of O(NTlogT). Second, estimating the covariance matrix requires computing all pairwise cross-products among the samples, which has complexity *O*(*NT*^2^). Finally, an eigenvalue decomposition is carried out on a T×T covariance matrix, where standard algorithms have a worst-case complexity of *O*(*T*^3^) [32]. Therefore, the overall time complexity of FPCA is $O(NT^{2}\;+\;T^{3})$. The method is highly sensitive to increases in *T*, since the cubic term dominates the computational cost. However, when *T* is fixed and *N* increases, the method remains relatively efficient. It may nonetheless become a bottleneck for long time-series data, and is thus more suitable for applications with time series of moderate length.

For the WLR method, the computation starts with forming continuous curves, a step that involves simple interpolation and has complexity *O*(*NT*). Its segmentation mechanism is based on detecting intersections between curves: for each of the N(N−1)/2 curve pairs, *T* time points are examined and sign changes are computed, giving a complexity of *O*(*N*^2^*T*). The subsequent steps of sorting intersections, removing duplicates, and generating segment boundaries are, in the worst case, still dominated by *O*(*N*^2^*T*). For each segment, sorting is performed based on the mean of *N* samples, with complexity O(NlogN)×S. The weighted scoring part has complexity *O*(*NS*). Therefore, the overall complexity of the WLR method is $O(NT\;+\;N^{2}T\;+\;N^{2}\log N\;+\;NS\log N\;+\;NS)$. When *S* is small, lower-order terms can be neglected and the complexity simplifies to $O(N^{2}T+NS\log N)$, indicating that WLR is computationally inefficient in scenarios where *N* is large.

For the h-MD method, the core computation lies in constructing the pairwise distance matrix between samples. According to the definition given earlier, computing the *L*^2^-norm distance between any two samples *X*_*i*_ and *X*_*j*_ requires traversing *T* time points, so the complexity of a single distance calculation is *O*(*T*). To obtain the depth ranking of all samples, an N×N pairwise distance matrix must be computed. Therefore, the overall time complexity of h-MD is *O*(*N*^2^*T*). This method is quite sensitive to increases in the sample size *N*, when *N* is large, its computational efficiency becomes relatively low.

For the RTD method, the randomized Tukey depth reduces dimensionality through projections, and its computation consists of two parts: projection and one-dimensional ordering. Let *V* be the number of random projection directions, and project the *N* samples onto these *V* directions. Each projection involves computing an inner product between *T*-dimensional vectors, so the complexity of a single projection is, and *O*(*T*) the total projection cost is *O*(*VNT*). For each projection direction, the univariate Tukey depth of the *N* projected points is computed. This typically requires sorting the projected values, and standard sorting algorithms have complexity O(NlogN). Over *V* directions, this step has a total complexity of O(VNlogN). Therefore, the overall time complexity of RTD is O(V(NT+NlogN)). Since *V* is treated as a constant parameter, the complexity of RTD is mainly dominated by the *NT* term. Compared with h-MD, RTD offers a clear computational advantage in scenarios where *N* is large.

For the proposed FP-OWA method, the computational pipeline proceeds as follows. First, in the spline smoothing step we use GCV to select the smoothing parameter *λ* [33]. The overall complexity of this step is O(NTlogT+Nlogλ). Since PCA is then performed in a low-dimensional feature space, its cost *O*(*T*) is comparatively negligible. Second, DBSCAN clustering is applied to the feature sequence at *T* time points. With spatial indexing, the average-case complexity is O(TlogT), for dense data, however, the complexity degrades to *O*(*T*^2^) [28]. Thus, this step in total costs $O(T^{2}\log T\;+\;T\log T)$. As it is computed only once, it usually does not dominate the overall runtime, but it does introduce variability in the number of segments *S*. Subsequently, the MBD weighting step computes the pairwise band depth of the *N* curves within each segment. This requires exhaustive checking at *L* grid points(L≈T/S), with complexity $O(SN^{2}L)=O(N^{2}T)$ [19]. In this paper, we instead adopt a fast MBD variant based on stack-ranking [34], which reduces the per-time-point cost from quadratic to quasi-linear, O(NlogN). Consequently, over all *S* segments, computing the depth weights and performing integration and ranking has an overall complexity of O(S(T/S)·NlogN+NlogN)≈O(TNlogN). Meanwhile, variability in the DBSCAN-based segmentation leads to a *U*-shaped runtime curve: a larger *S* increases the ranking overhead, but at the same time shortens *L* and thereby reduces the MBD cost within each segment, which can ultimately improve overall efficiency.

## 3 Asymptotic property

In this section, we establish the asymptotic properties of the proposed adaptive functional piecewise ranking–weighting (FP-OWA) method through Theorems 1–5. We begin by stating the assumptions required for the theoretical analysis of the estimator.

A1: The true functions *f*_*i*_(*t*) belong to the Sobolev space *W*^*m*,2^[0,1] with m≥2, so that their derivatives up to order *m* are square–integrable, that is, ${\left\|f_{i}\right\|}_{W^{m,2}}={\left(\int_{0}^{1}{\left|f_{i}(t)\right|}^{2}dt+\int_{0}^{1}{\left|f_{i}^{\left(m\right)}(t)\right|}^{2}dt\right)}^{1/2}<\infty$

A2: The noise terms $\varepsilon_{it}$ are i.i.d with $E(\varepsilon_{ij})=0$ and $Var(\varepsilon_{ij})=\delta^{2}<\infty$, and they have bounded moments.

A3: The observation points *t*_*j*_ lie in [0,1] and are either uniformly distributed or satisfy a minimum–spacing condition: there exists a constant *c* > 0 such that $\min_{j}(t_{j+1}-t_{j})\geq c/T$, where *T* is the number of observation points.

A4: For each true change-point $\tau_{k}$ of the standardized data, there exists a constant δ>0 such that within a neighbourhood $|\tau -\tau_{k}|<\eta$ the density p(τ) exhibits a jump, $|p(\tau_{k}^{+})-p(\tau_{k}^{-})|>\delta$.

A5: Within each segment the functions are Hölder continuous: there exist constantst α>0 and *L* > 0 such that, for all *t*, *s* in the same segment $|f_{i}(t)-f_{i}(s)|\leq L|t-s{|}^{\alpha }$. Finite jumps (discontinuities) are allowed at the segment boundaries, so that the smoothing procedure does not blur the change-points.

A6: The clustering parameters satisfy $\epsilon ≍T^{-1/\xi }$ and minPts=O(ζ), where ζ denotes the data dimension.

A7: The collection of functions {*f*_*i*_} is bounded in a compact subset ℱ⊂C[0,1] of the continuous function space, and the measure *ψ* is the Lebesgue measure.

A8: The band- depth kernel *h*(*f*,*g*) is bounded and Lipschitz continuous: there exists *M* > 0 such that |h(f,g)|≤M and *h* satisfies a Lipschitz condition.

A9: For any i≠land any segment $\left[a_{k},b_{k}\right]$, $\int_{a_{k}}^{b_{k}}f_{i}(t)dt\neq \int_{a_{k}}^{b_{k}}f_{l}(t)dt$.

A10: The number of segmentations is finite K<∞, and the weight w are positive and sum to one.

Note 1: Assumption A1 ensures that the roughness penalty term is well-defined and bounded. Assumption A2 provides the moment conditions needed to apply the law of large numbers and Bernstein-type inequalities. Assumption A3 guarantees that the condition number of the design matrix remains bounded, preventing ill-conditioning of the spline basis in regions with sparse observations. Assumption A4 is a regularity condition required for the convergence of DBSCAN. Assumption A5 enforces within-segment smoothness so that local oscillations do not interfere with density estimation. Assumption A6 specifies the asymptotic rate of the DBSCAN tuning parameters and ensures that adapts to the sample size, thereby avoiding both missed boundaries and unintended merging of segments. Assumption A7 restricts the function space so that the associated U-statistics are well-defined, while the use of the Lebesgue measure guarantees that the depth computation is measure-invariant. Assumption A8 provides variance control for the U-process and justifies the use of Hoeffding-type inequalities. Assumption A9 prevents discontinuities of the ranking functional without imposing parallel-curve assumptions and ensures the applicability of Slutsky’s lemma. Finally, Assumption A10 (finite segmentation) prevents divergence of infinite sums and guarantees the consistency of the aggregated score.

**Theorem 1:** Suppose Assumptions A1–A3 hold and consider the functional data model $y_{i}(t_{j})=f_{i}(t_{j})\;+\;\varepsilon_{i}(t_{j})$. Let ${\hat{f}}_{i}(t)$ be the estimator obtained by expanding in a spline basis with a roughness penalty. Then, as n→∞,


$$\sup_{t\in [0,T]}\left|{\hat{f}}_{i}(t)-f_{i}(t)\right|\overset{p\;}{\to }0,$$

where, *n* is the number of observation points and the smoothing parameter *lambda* satisfies λ→0 and nλ→∞.

Theorem 1 shows that, under Assumptions A1–A3, the penalized spline estimator ${\hat{f}}_{i}(t)$ converges uniformly in probability to the true function *f*_*i*_(*t*) as the number of observation points *n* increases. Thus, the smoothing step effectively removes noise without distorting the underlying functional trend, providing a sound basis for the accuracy of the subsequent ranking procedure.

**Theorem 2:** Suppose Assumptions A4–A6 hold. For the standardized functional data


$$z_{i}(t)=\frac{y_{i}(t)-\overset{―}{y}(t)}{\sigma (t)},$$

let $T=t_{1},t_{2},...,t_{m}$ denote the set of true change-points. When the sample size is sufficiently large and the DBSCAN parameters *ε* and minPts are chosen appropriately, the set of estimated change-points ${\hat{T}}_{n}={\hat{t}}_{1},{\hat{t}}_{2},...,{\hat{t}}_{m}$ obtained from the DBSCAN-based automatic segmentation satisfies, as n→∞,


$$P(H(T,{\hat{T}}_{n})<\delta )\to 1,$$

where *H* denotes the Hausdorff distance and *δ* is arbitrary.

Theorem 2 indicates that, under Assumptions A4–A6, the automatic segmentation produced by DBSCAN is consistent: the Hausdorff distance between the estimated and true sets of segment boundaries converges to zero as the sample size grows. This means that the segmentation accurately captures local structural changes in the data, thereby avoiding subjectivity and bias in determining the segment boundaries.

**Theorem 3:** Suppose Assumptions A7–A8 hold. For the collection of functions $f_{1}(t),f_{2}(t),...,f_{n}(t)$, the sample modified band depth *MBD*_*n*_(*f*) is a uniformly strongly consistent estimator of the population band depth *MBD*(*f*), that is, as n→∞,


$$\sup_{f\in ℱ}|MBD_{n}(f)-MBD(f)|\overset{a.s.\;}{\to }0,$$

where ℱ represents the function space under consideration.

**Theorem 4:** Suppose Assumptions A1–A10 hold. For each curve *f*_*i*_(*t*), define $I_{j}(f_{i})=\int_{t_{j}}^{t_{j+1}}f_{i}(t)dt$ as the integral of *f*_*i*_(*t*) over the *j*-th segment. Let $R_{j}(f_{i})$ denote the true rank of *f*_*i*_(*t*) on this segment, and let ${\hat{R}}_{j}(f_{i})$ be the rank computed from the estimated function ${\hat{f}}_{i}(t)$. Then, as n→∞,


$$P({\hat{R}}_{j}(f_{i})=R_{j}(f_{i}))\to 1,$$

so that the estimated ranks converge to the true segment-wise ranks.

**Theorem 5:** Suppose Assumptions A1–A10 hold. Define the true overall score of curve *f*_*i*_ by $S(f_{i})=\sum_{j=1}^{m}R_{j}(f_{i})$ and the estimated overall score by $\hat{S}(f_{i})=\sum_{j=1}^{\hat{m}}{\hat{w}}_{j}{\hat{R}}_{j}(f_{i})$. Then, as n→∞,


$$\hat{S}(f_{i})\overset{p\;}{\to }S(f_{i}).$$

Moreover, the ranking induced by $\hat{S}(f_{i})$ converge to the true induced by *S*(*f*_*i*_).

Theorem 3 shows that, under Assumptions A1–A10, the estimator of the modified band depth (MBD) is consistent. Theorems 4 and 5 further establish that both the estimated within-segment ranks and the global aggregated scores converge to their true counterparts. Taken together, these results provide theoretical justification that, even in the presence of noise, missing values, or outliers, the FP-OWA method yields stable rankings that faithfully reflect the true relative ordering of the sample curves.

Note 2: Detailed proofs of Theorems 1–5 are provided in the S1 Appendix.

## 4 Simulation study

To evaluate the performance of the proposed FP-OWA method for ranking functional data, we conduct a Monte Carlo simulation study. Specifically, we perform 500 independent replications to compare five methods—FPCA, WLR, h-MD, RTD, and FP-OWA—in terms of ranking accuracy and robustness under different noise settings.

### 4.1 Generation of simulated data

The functional observations are generated according to

$$f_{i}(t)=s_{i}t+\sin(2\pi (t+p_{i}))+\varepsilon_{i}(t),$$ (11)

where $s_{i}\sim U(3.5,4.5)$ denotes the slope of a randomly generated linear trend component, $p_{i}\sim U(0,0.2)$ is a random phase shift of the periodic component, and $\varepsilon_{i}(t)$ represents the noise term. To mimic disturbances that arise in practical applications, we design five noise schemes based on this baseline model:

(1) Gaussian noise: Independent Gaussian perturbations with mean μ=0 and standard deviations δ=0.01, 0.1, and 0.5are added to the simulated curves. This setting examines how well the methods adapt to conventional Gaussian disturbances.

(2) Spike noise: To model sporadic extreme measurements, we randomly select 1%, 10%, and 30% of the sampling points and inflate their original values by a factor of 5, thereby assessing the ability of the methods to handle abrupt outliers.

(3) Amplitude noise: Localized sharp oscillations are created by amplifying the signal magnitude at randomly chosen 30%, 50%, and 80% of the time points, which is used to test how the methods deal with pronounced local changes.

(4) Poisson noise: Poisson-distributed perturbations with rate parameters λ=0.01, 0.1 and 0.3 are superimposed to emulate discrete event noise, allowing us to evaluate robustness against low-frequency but high-intensity discrete shocks.

(5) Laplace noise: Heavy-tailed Laplace perturbations with mean 0 and scale parameters 0.01, 0.1, and 0.3 are added. Compared with Gaussian noise, the heavier tails provide a more stringent test of the methods’ tolerance to extreme values.

**Fig 1:**
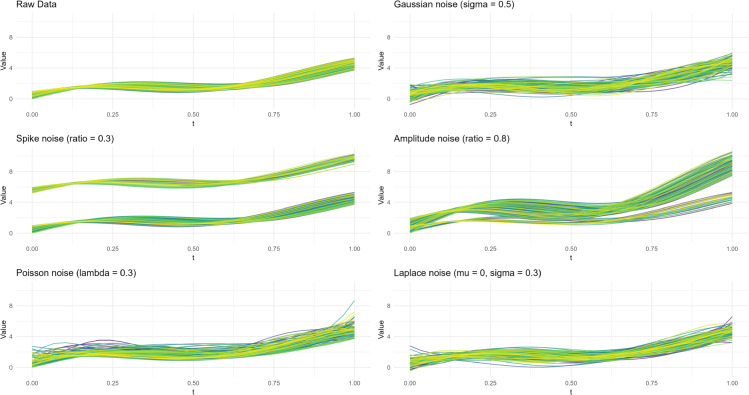
Schematic diagram of the original data and noise data.

The original data-generating process, along with the five noise-contaminated variants, is visualized in Fig 1.

### 4.2 Evaluation criteria

To compare the ranking accuracy and robustness of the five methods—FPCA, WLR, h-MD, RTD and FP-OWA—we adopt the following three criteria:

(1) Kendall’s Tau Test: We compute Kendall’s tau by comparing all possible pairs of observations and evaluating the proportion of concordant versus discordant pairs:

$$\tau =\frac{2(P-Q)}{n(n-1)},$$ (12)

where *P* is the number of concordant pairs, i.e., pairs (*i*, *j*), for which the relative order of the two observations is the same in both rankings, and *Q* is the number of discordant pairs, i.e., pairs for which the relative order is reversed. Values of *τ* closer to 1 indicate higher agreement between the rankings.

(2) Spearman’s Rho test: The original scores are first converted to ranks, and then the Pearson correlation coefficient between the two rank sequences is computed as

$$\rho =1-\frac{6\sum_{i=1}^{n}d_{i}^{2}}{n(n^{2}-1)},$$ (13)

where *d*_*i*_ denotes the difference between the two ranks for observation *i*. Values of *ρ* closer to 1 correspond to stronger rank correlation.

(3) Mean Absolute Error (MAE): MAE is defined as the average absolute difference between the predicted scores and the true integral scores. Smaller MAE values indicate that the predicted scores are closer to the true scores and thus reflect better ranking accuracy.

$$MAE=\frac{1}{n}\sum_{i=1}^{n}\left|\hat{S}\left(f_{i}\right)-S\left(f_{i}\right)\right|.$$ (14)

### 4.3 Simulation results and analysis

For each simulated data set, we apply FPCA, WLR, h-MD, RTD and FP-OWA to obtain the corresponding rankings. The resulting rankings are then compared with those obtained from the data without added white noise, and the three evaluation criteria are computed. Tables 1–5 report the simulation results under Gaussian white noise, Spike noise, Amplitude noise, Poisson noise and Laplace noise, respectively.

**Table 1:** Performance of five methods under Gaussian white noise.

n	t	Noise level	Sorting method	Kendall’s Tau	Spearman’s Rho	MAE	Run time
100	30	0.01	FPCA	0.1962	0.2844	0.1326	0.0265
				(0.1874, 0.2051)	(0.2718, 0.2971)	(0.1312, 0.1340)	
			WLR	0.7082	0.8815	0.0533	0.8798
				(0.7047, 0.7117)	(0.8789, 0.8841)	(0.0526, 0.0539)	
			h-MD	0.0091	0.0043	0.1678	0.1657
				(0.0001, 0.0182)	(–0.0087, 0.0174)	(0.1661, 0.1695)	
			RTD	0.0369	0.0466	0.1585	0.1527
				(0.0305, 0.0433)	(0.0381, 0.0550)	(0.1575, 0.1595)	
			FP-OWA	0.7968	0.9407	0.0392	0.1653
				(0.7935, 0.8001)	(0.9387, 0.9427)	(0.0386, 0.0398)	
		0.1	FPCA	0.1952	0.2832	0.1317	0.0259
				(0.1863, 0.2042)	(0.2704, 0.2960)	(0.1302, 0.1331)	
			WLR	0.6487	0.8434	0.0619	0.9510
				(0.6454, 0.6520)	(0.8405, 0.8462)	(0.0614, 0.0625)	
			h-MD	0.0086	0.0066	0.1646	0.1546
				(0.0005, 0.0167)	(–0.0052, 0.0185)	(0.1631, 0.1661)	
			RTD	0.0276	0.0373	0.1663	0.1461
				(0.0231, 0.0322)	(0.0308, 0.0438)	(0.1654, 0.1672)	
			FP-OWA	0.7045	0.8831	0.0536	0.1572
				(0.6984, 0.7105)	(0.8783, 0.8879)	(0.0526, 0.0546)	
		0.5	FPCA	0.1754	0.2535	0.1274	0.0260
				(0.1607, 0.1900)	(0.2325, 0.2745)	(0.1255, 0.1293)	
			WLR	0.3898	0.5569	0.0996	1.3726
				(0.3849, 0.3947)	(0.5504, 0.5634)	(0.0990, 0.1003)	
			h-MD	0.0014	0.0016	0.1551	0.1612
				(–0.0047, 0.0075)	(–0.0073, 0.0106)	(0.1540, 0.1561)	
			RTD	0.0024	0.0036	0.1767	0.1500
				(–0.0028, 0.0076)	(–0.0039, 0.0112)	(0.1757, 0.1777)	
			FP-OWA	0.3949	0.5600	0.0989	0.1626
				(0.3875, 0.4022)	(0.5505, 0.5696)	(0.0979, 0.0999)	
300	50	0.01	FPCA	0.1923	0.2812	0.1314	0.0310
				(0.1871, 0.1975)	(0.2737, 0.2887)	(0.1306, 0.1323)	
			WLR	0.7108	0.8874	0.0530	58.9628
				(0.7089, 0.7128)	(0.8860, 0.8888)	(0.0527, 0.0534)	
			h-MD	0.0098	0.0046	0.1694	1.0797
				(0.0041, 0.0155)	(–0.0036, 0.0128)	(0.1684, 0.1704)	
			RTD	0.0356	0.0447	0.1580	0.3365
				(0.0291, 0.0422)	(0.0362, 0.0531)	(0.1571, 0.1589)	
			FP-OWA	0.8036	0.9498	0.0369	0.2774
				(0.8024, 0.8048)	(0.9492, 0.9504)	(0.0367, 0.0372)	
		0.1	FPCA	0.1879	0.2758	0.1304	0.0308
				(0.1826, 0.1932)	(0.2680, 0.2835)	(0.1296, 0.1312)	
			WLR	0.6500	0.8491	0.0614	70.3298
				(0.6483, 0.6518)	(0.8476, 0.8506)	(0.0611, 0.0617)	
			h-MD	0.0077	0.0048	0.1650	1.0868
				(0.0026, 0.0128)	(–0.0027, 0.0124)	(0.1640, 0.1659)	
			RTD	0.0279	0.0381	0.1670	0.3364
				(0.0241, 0.0316)	(0.0327, 0.0434)	(0.1664, 0.1677)	
			FP-OWA	0.7573	0.9276	0.0437	0.2765
				(0.7561, 0.7585)	(0.9269, 0.9283)	(0.0435, 0.0439)	
		0.5	FPCA	0.0965	0.1420	0.1339	0.0310
				(0.0849, 0.1082)	(0.1249, 0.1591)	(0.1325, 0.1353)	
			WLR	0.3912	0.5628	0.0989	103.0949
				(0.3885, 0.3939)	(0.5592, 0.5664)	(0.0985, 0.0992)	
			h-MD	0.0016	0.0020	0.1536	1.0699
				(–0.0021, 0.0052)	(–0.0034, 0.0074)	(0.1529, 0.1542)	
			RTD	0.0017	0.0023	0.1791	0.3345
				(–0.0014, 0.0047)	(–0.0022, 0.0068)	(0.1784, 0.1797)	
			FP-OWA	0.4389	0.6216	0.0923	0.2799
				(0.4340, 0.4438)	(0.6153, 0.6280)	(0.0917, 0.0930)	

**Table 2:** Performance of five methods under Spike noise.

n	t	Noise level	Sorting method	Kendall’s Tau	Spearman’s Rho	MAE	Run time
100	30	0.01	FPCA	0.9244	0.9730	0.2211	0.0247
				(0.9211, 0.9277)	(0.9713, 0.9747)	(0.2199, 0.2222)	
			WLR	0.6951	0.8657	0.0844	0.5014
				(0.6849, 0.7053)	(0.8573, 0.8740)	(0.0816, 0.0873)	
			h-MD	0.0097	0.0057	0.1751	0.1674
				(0.0004, 0.0189)	(–0.0075, 0.0189)	(0.1736, 0.1767)	
			RTD	0.0574	0.0833	0.1555	0.1519
				(0.0510, 0.0639)	(0.0749, 0.0916)	(0.1545, 0.1565)	
			FP-OWA	0.7801	0.9209	0.0571	0.1649
				(0.7769, 0.7834)	(0.9185, 0.9233)	(0.0549, 0.0592)	
		0.1	FPCA	0.8081	0.8196	0.2228	0.0225
				(0.8049, 0.8114)	(0.8148, 0.8243)	(0.2217, 0.2239)	
			WLR	0.5823	0.7239	0.1093	0.6696
				(0.5781, 0.5866)	(0.7188, 0.7291)	(0.1084, 0.1102)	
			h-MD	0.0085	0.0033	0.1795	0.1532
				(0.0004, 0.0167)	(–0.0091, 0.0156)	(0.1782, 0.1809)	
			RTD	0.2042	0.2944	0.1412	0.1391
				(0.1978, 0.2106)	(0.2852, 0.3036)	(0.1402, 0.1422)	
			FP-OWA	0.6530	0.7710	0.0830	0.1541
				(0.6489, 0.6571)	(0.7661, 0.7760)	(0.0808, 0.0851)	
		0.3	FPCA	0.5748	0.5815	0.2224	0.0200
				(0.5703, 0.5793)	(0.5747, 0.5883)	(0.2212, 0.2236)	
			WLR	0.4109	0.5127	0.1477	0.4325
				(0.4058, 0.4160)	(0.5057, 0.5198)	(0.1465, 0.1488)	
			h-MD	0.0017	–0.0025	0.1711	0.1303
				(–0.0056, 0.0090)	(–0.0137, 0.0086)	(0.1698, 0.1725)	
			RTD	0.1897	0.2490	0.1405	0.1226
				(0.1827, 0.1968)	(0.2395, 0.2585)	(0.1393, 0.1417)	
			FP-OWA	0.4630	0.5476	0.1251	0.1308
				(0.4582, 0.4678)	(0.5407, 0.5544)	(0.1232, 0.1271)	
300	50	0.01	FPCA	0.9427	0.9777	0.2240	0.0273
				(0.9406, 0.9449)	(0.9768, 0.9786)	(0.2233, 0.2246)	
			WLR	0.6992	0.8711	0.0836	57.1630
				(0.6972, 0.7012)	(0.8695, 0.8728)	(0.0831, 0.0841)	
			h-MD	0.0130	0.0095	0.1778	1.0667
				(0.0077, 0.0183)	(0.0018, 0.0172)	(0.1769, 0.1787)	
			RTD	0.0612	0.0887	0.1545	0.3338
				(0.0548, 0.0676)	(0.0805, 0.0969)	(0.1537, 0.1554)	
			FP-OWA	0.7884	0.9312	0.0411	0.2772
				(0.7871, 0.7897)	(0.9302, 0.9323)	(0.0408, 0.0413)	
		0.1	FPCA	0.8135	0.8206	0.2255	0.0273
				(0.8117, 0.8154)	(0.8178, 0.8233)	(0.2249, 0.2262)	
			WLR	0.5839	0.7284	0.1091	49.1462
				(0.5814, 0.5863)	(0.7253, 0.7313)	(0.1085, 0.1096)	
			h-MD	0.0094	0.0048	0.1809	1.1160
				(0.0044, 0.0144)	(–0.0029, 0.0124)	(0.1800, 0.1818)	
			RTD	0.2039	0.2955	0.1404	0.3453
				(0.1980, 0.2097)	(0.2872, 0.3038)	(0.1395, 0.1413)	
			FP-OWA	0.6596	0.7794	0.0675	0.2847
				(0.6575, 0.6617)	(0.7766, 0.7822)	(0.0670, 0.0680)	
		0.3	FPCA	0.5758	0.5791	0.2261	0.0251
				(0.5732, 0.5784)	(0.5752, 0.5830)	(0.2254, 0.2267)	
			WLR	0.4112	0.5132	0.1484	32.3931
				(0.4084, 0.4140)	(0.5092, 0.5171)	(0.1478, 0.1490)	
			h-MD	0.0051	0.0019	0.1697	1.0046
				(0.0010, 0.0091)	(–0.0043, 0.0082)	(0.1690, 0.1705)	
			RTD	0.1951	0.2576	0.1402	0.3099
				(0.1893, 0.2008)	(0.2498, 0.2653)	(0.1392, 0.1412)	
			FP-OWA	0.4650	0.5496	0.1164	0.2522
				(0.4624, 0.4677)	(0.5457, 0.5535)	(0.1158, 0.1171)	

**Table 3:** Performance of five methods under Amplitude noise.

n	t	Noise level	Sorting method	Kendall’s Tau	Spearman’s Rho	MAE	Run time
100	30	0.3	FPCA	0.4364	0.5363	0.1792	0.0221
				(0.4313, 0.4414)	(0.5293, 0.5433)	(0.1781, 0.1803)	
			WLR	0.4008	0.5014	0.1447	0.4695
				(0.3957, 0.4058)	(0.4944, 0.5084)	(0.1436, 0.1458)	
			h-MD	0.0049	0.0014	0.1863	0.1298
				(–0.0019, 0.0117)	(–0.0092, 0.0121)	(0.1850, 0.1876)	
			RTD	0.2550	0.3374	0.1320	0.1207
				(0.2492, 0.2609)	(0.3297, 0.3452)	(0.1310, 0.1330)	
			FP-OWA	0.4627	0.5470	0.1247	0.1288
				(0.4579, 0.4676)	(0.5401, 0.5538)	(0.1229, 0.1266)	
		0.5	FPCA	0.3701	0.4544	0.1705	0.0258
				(0.3647, 0.3754)	(0.4468, 0.4621)	(0.1695, 0.1716)	
			WLR	0.3338	0.4193	0.1584	0.5608
				(0.3284, 0.3392)	(0.4116, 0.4271)	(0.1572, 0.1595)	
			h-MD	0.0034	0.0012	0.1667	0.1627
				(–0.0025, 0.0093)	(–0.0075, 0.0098)	(0.1656, 0.1678)	
			RTD	0.0997	0.1305	0.1489	0.1472
				(0.0924, 0.1071)	(0.1209, 0.1402)	(0.1478, 0.1500)	
			FP-OWA	0.3899	0.4613	0.1423	0.1621
				(0.3843, 0.3954)	(0.4535, 0.4691)	(0.1406, 0.1440)	
		0.8	FPCA	0.4884	0.6092	0.1514	0.0265
				(0.4838, 0.4931)	(0.6030, 0.6155)	(0.1504, 0.1523)	
			WLR	0.4654	0.5832	0.1308	0.6820
				(0.4607, 0.4701)	(0.5770, 0.5895)	(0.1299, 0.1318)	
			h-MD	0.0048	0.0002	0.1701	0.1639
				(–0.0030, 0.0126)	(–0.0117, 0.0121)	(0.1687, 0.1715)	
			RTD	–0.1455	–0.1957	0.1921	0.1511
				(–0.1561, -0.1348)	(–0.2105, -0.1810)	(0.1903, 0.1938)	
			FP-OWA	0.5358	0.6325	0.1065	0.1622
				(0.5313, 0.5402)	(0.6264, 0.6386)	(0.1048, 0.1083)	
300	50	0.3	FPCA	0.4347	0.5357	0.1792	0.0293
				(0.4318, 0.4375)	(0.5318, 0.5397)	(0.1787, 0.1798)	
			WLR	0.4012	0.5018	0.1453	40.4009
				(0.3984, 0.4040)	(0.4979, 0.5058)	(0.1447, 0.1459)	
			h-MD	0.0066	0.0040	0.1867	1.0705
				(0.0029, 0.0104)	(–0.0019, 0.0098)	(0.1859, 0.1874)	
			RTD	0.2596	0.3465	0.1324	0.3230
				(0.2551, 0.2640)	(0.3407, 0.3522)	(0.1316, 0.1331)	
			FP-OWA	0.4651	0.5496	0.1164	0.2679
				(0.4624, 0.4677)	(0.5457, 0.5535)	(0.1158, 0.1170)	
		0.5	FPCA	0.3729	0.4596	0.1686	0.0291
				(0.3697, 0.3761)	(0.4550, 0.4642)	(0.1681, 0.1692)	
			WLR	0.3392	0.4265	0.1583	36.4461
				(0.3361, 0.3424)	(0.4219, 0.4311)	(0.1577, 0.1590)	
			h-MD	0.0074	0.0067	0.1654	1.0825
				(0.0039, 0.0110)	(0.0015, 0.0120)	(0.1648, 0.1661)	
			RTD	0.1099	0.1463	0.1488	0.3301
				(0.1034, 0.1165)	(0.1377, 0.1548)	(0.1480, 0.1496)	
			FP-OWA	0.3997	0.4718	0.1340	0.2736
				(0.3965, 0.4028)	(0.4672, 0.4764)	(0.1333, 0.1346)	
		0.8	FPCA	0.4892	0.6146	0.1491	0.0283
				(0.4865, 0.4919)	(0.6108, 0.6183)	(0.1486, 0.1496)	
			WLR	0.4724	0.5924	0.1306	45.4214
				(0.4697, 0.4751)	(0.5887, 0.5961)	(0.1301, 0.1312)	
			h-MD	0.0063	0.0030	0.1694	1.0678
				(0.0017, 0.0109)	(–0.0041, 0.0101)	(0.1686, 0.1702)	
			RTD	–0.1315	–0.1774	0.1902	0.3287
				(–0.1420, -0.1211)	(–0.1921, -0.1628)	(0.1886, 0.1917)	
			FP-OWA	0.5451	0.6438	0.0953	0.2710
				(0.5426, 0.5476)	(0.6402, 0.6473)	(0.0947, 0.0959)	

**Table 4:** Performance of five methods under Poisson noise.

n	t	Noise level	Sorting method	Kendall’s Tau	Spearman’s Rho	MAE	Run time
100	30	0.01	FPCA	0.1915	0.2788	0.1321	0.0261
				(0.1830, 0.2000)	(0.2667, 0.2910)	(0.1308, 0.1335)	
			WLR	0.6899	0.8675	0.0565	0.8909
				(0.6865, 0.6933)	(0.8649, 0.8701)	(0.0558, 0.0571)	
			h-MD	0.0199	0.0167	0.1684	0.1600
				(0.0112, 0.0286)	(0.0040, 0.0294)	(0.1669, 0.1699)	
			RTD	0.0633	0.0858	0.1581	0.1468
				(0.0581, 0.0686)	(0.0788, 0.0927)	(0.1572, 0.1589)	
			FP-OWA	0.7642	0.9179	0.0467	0.0410
				(0.7608, 0.7675)	(0.9154, 0.9203)	(0.0459, 0.0476)	
		0.1	FPCA	0.1688	0.2459	0.1308	0.0259
				(0.1575, 0.1801)	(0.2296, 0.2622)	(0.1293, 0.1323)	
			WLR	0.5579	0.7511	0.0780	1.0117
				(0.5541, 0.5616)	(0.7470, 0.7551)	(0.0773, 0.0786)	
			h-MD	0.0321	0.0284	0.1622	0.1559
				(0.0251, 0.0391)	(0.0179, 0.0390)	(0.1609, 0.1634)	
			RTD	0.1380	0.1921	0.1601	0.1445
				(0.1329, 0.1430)	(0.1847, 0.1996)	(0.1592, 0.1610)	
			FP-OWA	0.5692	0.7541	0.0778	0.1578
				(0.5636, 0.5748)	(0.7477, 0.7606)	(0.0767, 0.0790)	
		0.3	FPCA	0.1707	0.2456	0.1312	0.0242
				(0.1550, 0.1863)	(0.2233, 0.2680)	(0.1294, 0.1330)	
			WLR	0.4109	0.5831	0.0971	1.1961
				(0.4064, 0.4153)	(0.5772, 0.5889)	(0.0965, 0.0978)	
			h-MD	0.0279	0.0307	0.1612	0.1546
				(0.0215, 0.0344)	(0.0211, 0.0403)	(0.1601, 0.1624)	
			RTD	0.1210	0.1717	0.1614	0.1424
				(0.1153, 0.1266)	(0.1636, 0.1798)	(0.1604, 0.1624)	
			FP-OWA	0.4033	0.5708	0.0988	0.1549
				(0.3968, 0.4099)	(0.5623, 0.5792)	(0.0973, 0.0993)	
300	50	0.01	FPCA	0.1849	0.2710	0.1304	0.0312
				(0.1795, 0.1904)	(0.2631, 0.2789)	(0.1295, 0.1312)	
			WLR	0.6918	0.8727	0.0566	61.6932
				(0.6898, 0.6939)	(0.8712, 0.8742)	(0.0562, 0.0571)	
			h-MD	0.0258	0.0238	0.1718	1.0981
				(0.0207, 0.0309)	(0.0162, 0.0315)	(0.1708, 0.1727)	
			RTD	0.0657	0.0894	0.1570	0.3396
				(0.0610, 0.0703)	(0.0835, 0.0954)	(0.1562, 0.1577)	
			FP-OWA	0.7733	0.9315	0.0417	0.2815
				(0.7719, 0.7746)	(0.9308, 0.9323)	(0.0414, 0.0420)	
		0.1	FPCA	0.1442	0.2127	0.1298	0.0311
				(0.1356, 0.1529)	(0.2000, 0.2254)	(0.1287, 0.1309)	
			WLR	0.5584	0.7560	0.0787	76.4275
				(0.5563, 0.5604)	(0.7539, 0.7582)	(0.0783, 0.0791)	
			h-MD	0.0364	0.0352	0.1626	1.0794
				(0.0324, 0.0404)	(0.0291, 0.0412)	(0.1618, 0.1633)	
			RTD	0.1407	0.1985	0.1616	0.3341
				(0.1372, 0.1442)	(0.1935, 0.2035)	(0.1610, 0.1623)	
			FP-OWA	0.6048	0.8033	0.0691	0.2794
				(0.6026, 0.6070)	(0.8009, 0.8056)	(0.0686, 0.0695)	
		0.3	FPCA	0.1076	0.1573	0.1353	0.0311
				(0.0948, 0.1204)	(0.1387, 0.1759)	(0.1339, 0.1366)	
			WLR	0.4078	0.5840	0.0968	98.3301
				(0.4052, 0.4104)	(0.5806, 0.5873)	(0.0964, 0.0972)	
			h-MD	0.0361	0.0435	0.1597	1.1080
				(0.0324, 0.0399)	(0.0379, 0.0491)	(0.1591, 0.1604)	
			RTD	0.1273	0.1835	0.1642	0.3443
				(0.1237, 0.1309)	(0.1783, 0.1886)	(0.1635, 0.1648)	
			FP-OWA	0.4345	0.6157	0.0933	0.2851
				(0.4299, 0.4391)	(0.6097, 0.6216)	(0.0926, 0.0940)	

**Table 5:** Performance of five methods under Laplace noise.

n	t	Noise level	Sorting method	Kendall’s Tau	Spearman’s Rho	MAE	Run time
100	30	0.01	FPCA	0.1939	0.2822	0.1329	0.0252
				(0.1856, 0.2023)	(0.2702, 0.2941)	(0.1315, 0.1343)	
			WLR	0.7069	0.8812	0.0534	0.8587
				(0.7034, 0.7103)	(0.8787, 0.8837)	(0.0527, 0.0540)	
			h-MD	0.0097	0.0065	0.1666	0.1590
				(0.0001, 0.0193)	(–0.0071, 0.0201)	(0.1648, 0.1683)	
			RTD	0.0359	0.0459	0.1590	0.1477
				(0.0296, 0.0422)	(0.0376, 0.0542)	(0.1580, 0.1599)	
			FP-OWA	0.7951	0.9402	0.0393	0.1611
				(0.7919, 0.7984)	(0.9381, 0.9423)	(0.0387, 0.0399)	
		0.1	FPCA	0.1860	0.2720	0.1319	0.0258
				(0.1771, 0.1950)	(0.2592, 0.2848)	(0.1305, 0.1333)	
			WLR	0.6221	0.8209	0.0665	1.0471
				(0.6192, 0.6251)	(0.8181, 0.8236)	(0.0660, 0.0670)	
			h-MD	0.0044	0.0010	0.1622	0.1624
				(–0.0038, 0.0126)	(–0.0110, 0.0129)	(0.1608, 0.1637)	
			RTD	0.0235	0.0323	0.1685	0.1506
				(0.0191, 0.0278)	(0.0259, 0.0387)	(0.1677, 0.1694)	
			FP-OWA	0.6701	0.8560	0.0590	0.1640
				(0.6637, 0.6764)	(0.8505, 0.8615)	(0.0579, 0.0601)	
		0.3	FPCA	0.1536	0.2231	0.1298	0.0256
				(0.1401, 0.1671)	(0.2037, 0.2426)	(0.1281, 0.1315)	
			WLR	0.4442	0.6257	0.0927	1.2937
				(0.4400, 0.4485)	(0.6204, 0.6311)	(0.0921, 0.0934)	
			h-MD	–0.0052	–0.0086	0.1583	0.1622
				(–0.0117, 0.0012)	(–0.0181, 0.0010)	(0.1572, 0.1594)	
			RTD	0.0016	0.0022	0.1754	0.1496
				(–0.0036, 0.0068)	(–0.0054, 0.0099)	(0.1745, 0.1764)	
			FP-OWA	0.4482	0.6261	0.0914	0.1634
				(0.4407, 0.4557)	(0.6168, 0.6353)	(0.0903, 0.0924)	
300	50	0.01	FPCA	0.1887	0.2763	0.1317	0.0310
				(0.1833, 0.1941)	(0.2685, 0.2840)	(0.1309, 0.1326)	
			WLR	0.7080	0.8861	0.0533	58.6368
				(0.7060, 0.7100)	(0.8847, 0.8876)	(0.0529, 0.0537)	
			h-MD	0.0131	0.0090	0.1688	1.0638
				(0.0074, 0.0187)	(0.0008, 0.0172)	(0.1678, 0.1699)	
			RTD	0.0382	0.0490	0.1580	0.3294
				(0.0323, 0.0441)	(0.0412, 0.0567)	(0.1572, 0.1589)	
			FP-OWA	0.8017	0.9491	0.0372	0.2726
				(0.8005, 0.8029)	(0.9485, 0.9496)	(0.0370, 0.0374)	
		0.1	FPCA	0.1813	0.2668	0.1300	0.0302
				(0.1757, 0.1869)	(0.2587, 0.2749)	(0.1292, 0.1309)	
			WLR	0.6222	0.8252	0.0663	74.7591
				(0.6203, 0.6240)	(0.8235, 0.8269)	(0.0660, 0.0666)	
			h-MD	0.0094	0.0088	0.1619	1.0833
				(0.0046, 0.0142)	(0.0017, 0.0160)	(0.1611, 0.1628)	
			RTD	0.0224	0.0311	0.1698	0.3361
				(0.0194, 0.0254)	(0.0268, 0.0354)	(0.1691, 0.1704)	
			FP-OWA	0.7260	0.9075	0.0487	0.2755
				(0.7243, 0.7277)	(0.9062, 0.9088)	(0.0484, 0.0490)	
		0.3	FPCA	0.0995	0.1469	0.1338	0.0308
				(0.0887, 0.1103)	(0.1309, 0.1628)	(0.1325, 0.1351)	
			WLR	0.4432	0.6292	0.0924	101.6818
				(0.4408, 0.4457)	(0.6262, 0.6322)	(0.0920, 0.0927)	
			h-MD	0.0034	0.0045	0.1567	1.1070
				(–0.0004, 0.0072)	(–0.0012, 0.0102)	(0.1560, 0.1573)	
			RTD	0.0048	0.0069	0.1772	0.3399
				(0.0021, 0.0076)	(0.0027, 0.0110)	(0.1766, 0.1778)	
			FP-OWA	0.4952	0.6881	0.0841	0.2860
				(0.4900, 0.5004)	(0.6817, 0.6945)	(0.0834, 0.0849)	

As shown in Table 1, the FP-OWA method exhibits strong stability and high ranking consistency under all conditions. In particular, when the noise intensity varies, FP-OWA is able to maintain high accuracy and low error. In terms of overall performance, FP-OWA consistently achieves the highest values of Kendall’s tau and Spearman’s rho with relatively narrow confidence intervals, indicating a high level of agreement with the true ranking and excellent accuracy. At the same time, its run time is comparatively short, demonstrating a clear efficiency advantage over the other four methods. The WLR method attains medium levels of Kendall’s tau and Spearman’s rho, but its run time is substantially longer than that of FP-OWA and FPCA, and it increases sharply as the sample size grows, which is likely due to the high computational cost of its segmentation mechanism. The performance of FPCA in terms of Kendall’s tau and Spearman’s rho is relatively poor. A plausible explanation is that FPCA performs a global decomposition of variation, whereas in local integration tasks the weights of principal components should adapt to the underlying pattern of variation. In the present simulation design, multiple independent modes of variation are present, so ranking based solely on the first principal component score can be biased. For h-MD and RTD, the ranking consistency measures are close to zero. This is because both methods are designed to quantify centrality: curves with intermediate values attain the largest depth, while curves with the largest and smallest values lie on the periphery. As a result, depth-based methods cannot recover rankings that are defined purely in terms of magnitude. Overall, FP-OWA shows a clear advantage in ranking functional data contaminated by Gaussian white noise.

As shown in Table 2, the FP-OWA method still outperforms the other approaches when ranking functional data contaminated by spike noise. It is worth noting that although FPCA attains the highest rank-correlation coefficients under spike noise, its MAE is the largest among the five methods. This reflects the fact that, under the spike-noise design described earlier, the main differences among curves are captured in their overall amplitude, and FPCA is very effective at extracting such global shape features. However, while the first principal component can recover the relative ordering of the curves, it fails to accurately reproduce the underlying amplitude-based scores. In contrast, the proposed FP-OWA method achieves a more favorable balance: it maintains high rank consistency while attaining the lowest MAE among all competing methods. This indicates that FP-OWA not only preserves high ranking accuracy, but also provides more accurate numerical estimates. Combined with its relatively fast run time, FP-OWA is clearly superior to FPCA and the other four methods in terms of quantitative accuracy.

Amplitude noise differs from spike noise in that it perturbs the data amplitude more uniformly and is often used to mimic sensor instability or environmental interference. As shown in Table 3, the experimental results further confirm the overall superiority of the FP-OWA method. Compared with the WLR method based on local cross-information, the FPCA method based on variance maximization, and the depth-based h-MD and RTD methods, FP-OWA exhibits greater stability when dealing with large-amplitude perturbations in magnitude. More specifically, as the noise level increases to 0.8, FP-OWA not only maintains the lowest MAE, but also achieves higher ranking consistency measures than the other four methods. However, in contrast to the results under the other types of noise, Table 3 also shows that all methods perform relatively poorly in this setting. A plausible explanation is that the more uniformly distributed amplitude noise is intrinsically harder to handle than the abrupt spikes in spike noise. This suggests that future work could focus on further improving ranking methods specifically for such amplitude-noise scenarios.

As shown in Table 4, under Poisson noise the FPCA method performs poorly, as it cannot effectively accommodate the signal-dependent heteroscedasticity induced by the Poisson distribution; its ranking performance is markedly inferior to that of the h-MD and RTD methods and even breaks down in some scenarios. In contrast, FP-OWA exhibits excellent adaptivity, achieving the highest ranking concordance and the lowest mean absolute error across all noise levels. The advantage is particularly pronounced in terms of computational efficiency: when the sample size is *n* = 300 and the noise level is high, the WLR method is burdened by the computation of a large number of spurious crossing points, leading to a sharp increase in runtime, whereas FP-OWA, owing to its efficient feature aggregation algorithm, keeps the computation time below 0.3 seconds. This provides strong evidence for the stability of FP-OWA in handling Poisson-type functional data.

As shown in Table 5, under the experimental setting with Laplace noise contamination, the FP-OWA method demonstrates remarkable robustness for heavy-tailed data. The results indicate that the conventional FPCA method fails to accommodate the non-Gaussian characteristics of the data, leading to substantially lower ranking correlations. In contrast, benefiting from its distinctive aggregation mechanism, FP-OWA effectively suppresses the impact of impulsive noise and achieves the best ranking accuracy and the smallest MAE across all noise levels. More importantly, the local fluctuations induced by Laplace noise cause a dramatic increase in the computational complexity of the WLR method, whereas FP-OWA consistently maintains high computational efficiency, further confirming its effectiveness in handling non-Gaussian, heavy-tailed noise environments.

Taken together, the extensive simulation study under a variety of noise settings—including Gaussian, impulsive, amplitude, Poisson, and Laplace disturbances—demonstrates that the proposed FP-OWA method achieves substantially better overall performance than the prevailing approaches FPCA, WLR, h-MD, and RTD. Specifically, its advantages manifest in three main aspects. First, FP-OWA exhibits excellent ranking consistency and quantitative accuracy. Whether under standard Gaussian noise or more complex amplitude perturbations, FP-OWA consistently attains high ranking concordance and low MAE. In particular, in the presence of impulsive noise, FP-OWA simultaneously recovers the true ranking order and the underlying signal amplitudes with high precision, thereby overcoming the drawback of FPCA, which attains relatively high rank correlation at the cost of large estimation errors. At the same time, the experiments confirm that depth-based methods defined via centrality (h-MD, RTD) are not suitable for such amplitude-based ranking tasks. Second, FP-OWA is more robust to complex noise distributions. When confronted with the signal-dependent heteroscedasticity induced by Poisson noise and the heavy tails associated with Laplace noise, the variance-maximization-based FPCA method almost breaks down, whereas FP-OWA, leveraging its distinctive feature aggregation and smoothing mechanisms, effectively suppresses various forms of non-Gaussian noise and outliers, exhibiting strong adaptivity and stability across all complex noise settings. Finally, FP-OWA possesses superior computational scalability. In contrast to the WLR method, whose runtime grows almost exponentially when dealing with large sample sizes and high-frequency fluctuating noise due to the need to process a large number of local crossing points, FP-OWA consistently maintains high computational efficiency.

### 4.4 Sensitivity analysis

Considering that the performance of the method may depend on the two arguments *ε* and minPts of the DBSCAN clustering algorithm, this subsection uses the functional data generation process from Eq 11. Gaussian noise with μ=0 and δ=0.1 is added to simulate the perturbations in real data, and sensitivity analysis is conducted using the FP-OWA method. The k-NN distance plot is utilized to identify the elbow point, which helps in selecting the *ε* parameter. Furthermore, a sensitivity heatmap shows how the number of clusters changes with variations in *ε* and minPts, thereby guiding the selection of robust parameters.

**Fig 2:**
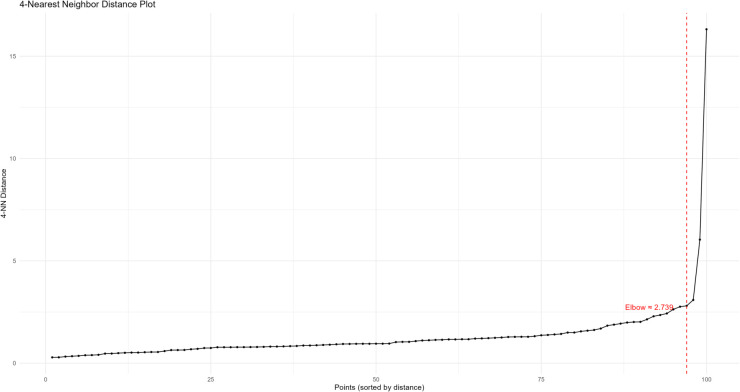
k-NN distance graph.

In Fig 2, the x-axis represents the indices of all data points sorted in ascending order by their “4-nearest neighbor distance,” while the y-axis shows the distance from each data point to its 4th nearest neighbor. The curve exhibits an inflection point where the slope shifts from gradual to steep, serving as the natural boundary between “core points” and “non-core points.” From the figure, it can be seen that the elbow occurs around 2.739, providing a reference range for selecting the *ε* parameter. The goal of the elbow plot is to identify the global distance boundary for “core points,” focusing only on the point density. This characteristic is particularly crucial when dealing with short sequences, highly seasonal data, or data with holiday (weekend) effects. Short sequences often suffer from sparse data due to the small sample size, and the elbow plot’s distance threshold can help exclude spurious sparse points caused by insufficient samples. Highly seasonal or holiday-affected data tend to form locally dense clusters at specific time points, and the global distance threshold of the elbow plot provides a foundational reference for such non-uniform dense structures, preventing misidentification of core points due to local fluctuations. However, the DBSCAN clustering result is determined by both *ε* and minPts. Especially in the case of the above-mentioned data features, a single *ε* threshold is insufficient to adapt to complex structures. Therefore, further plotting of the DBSCAN sensitivity heatmap is necessary to assist in selecting the optimal combination of parameters.

**Fig 3:**
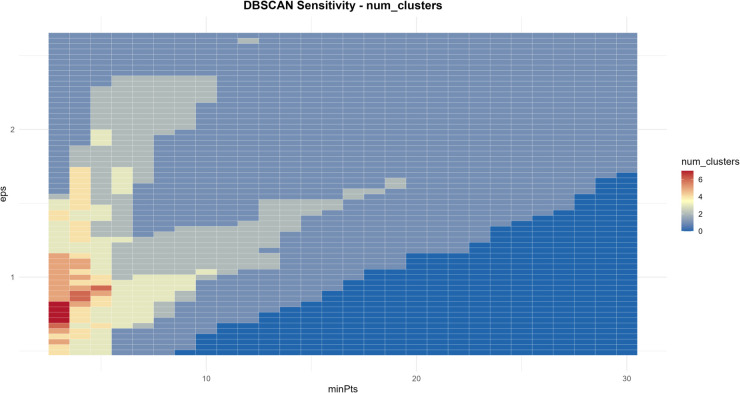
DBSCAN sensitivity heatmap.

From Fig 3, we can observe the changes in the number of clusters mapped across *ε* and minPts. In the lower-parameter red region, a high number of clusters is observed. This combination can accurately split sub-clusters of different seasons when dealing with highly seasonal data, but caution must be taken to avoid spurious cluster splitting in short sequences caused by an excessively small minPts. In the higher-parameter blue region, clusters tend to merge, reducing noise interference in short sequences, but potentially masking local special clusters related to holiday effects. Specifically, when *ε* is in the range of 0.8-1.5 and minPts is between 0-5, the heatmap shows a yellow-red tone corresponding to 4-6 clusters. This parameter range is most suitable for adapting to three types of special data. For short sequences, a moderate *ε* and smaller minPts combination can capture a limited number of core clusters while ensuring an adequate sample size. For highly seasonal data, this range helps distinguish independent clusters for different seasons while avoiding excessive merging within a cycle. For holiday (weekend) effect data, this range allows special patterns before and after holidays to be identified as independent sub-clusters, preventing confusion with regular data. When *ε* exceeds 2.3 or minPts exceeds 20, the heatmap predominantly shows blue, with the number of clusters reduced to 0-2. An excessively large *ε* can cause cross-cycle cluster merging in seasonal data, masking cycle differences, while a too-stringent minPts may misclassify valid short-sequence samples or local dense points related to holiday effects as noise, ultimately leading to cluster merging or failure. The above sensitivity analysis not only validates the reference value of the elbow plot for selecting *ε*, but also systematically enumerates multiple parameter combinations, providing a complete argument chain from single-threshold reference to optimal multi-parameter coordination for DBSCAN parameter selection. This ensures that the clustering results are both aligned with the inherent density of the data and meet the requirements of reasonable cluster numbers and structures, while also adapting to the special data characteristics of short sequences, high seasonality, and holiday (weekend) effects.

### 4.5 Performance of the FP-OWA method under missing data

**Fig 4:**
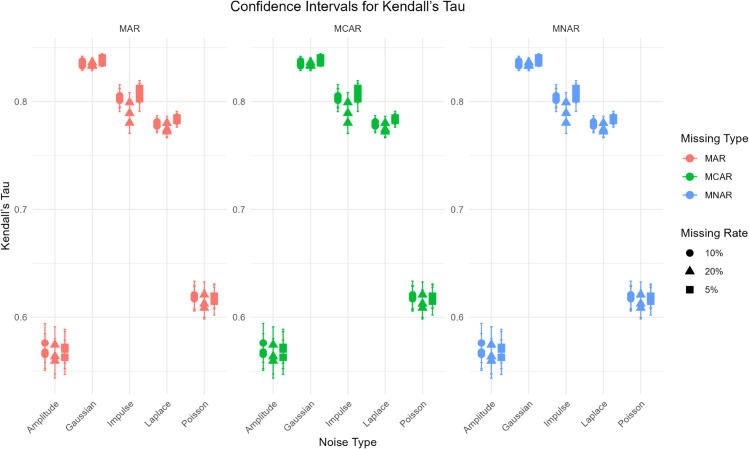
Performance of the FP-OWA method under different missing conditions and levels.

To validate the performance of the FP-OWA method in scenarios with missing data, we set *n* = 100 and *t* = 50 and adopt the functional data generation mechanism in Eq 11. Moderate levels of the aforementioned types of noise are added to mimic perturbations in real data. We then consider three missing-data mechanisms—missing completely at random (MCAR), missing at random (MAR), and missing not at random (MNAR)—and, for each mechanism, impose three missingness levels of 5%, 10%, and 20%. The results, assessed using the Kendall’s tau criterion, are reported in Fig 4.

As shown in Fig 4, regardless of the missing-data mechanism or the proportion of missingness, the method performs substantially better under Gaussian noise than under spike, amplitude, or Poisson noise, and the corresponding confidence intervals are noticeably narrower. From the perspective of missing-data mechanisms, the impact of the missing rate on Kendall’s tau is negligible under MCAR. For example, in the Gaussian-noise setting the Kendall’s tau values at 5%, 10%, and 20% missingness are 0.8360, 0.8376, and 0.8364, respectively, indicating that under completely random missingness the method can stably maintain ranking consistency. Under MAR, Kendall’s tau decreases slightly as the missing rate rises. In the spike-noise case, for instance, Kendall’s tau drops from 0.8121 at a 5% missing rate to 0.7893 at a 20% missing rate, reflecting the fact that MAR is related to the observed variables: the higher the missing rate, the more difficult it becomes to recover the lost information via interpolation, which in turn mildly affects ranking consistency. A similar pattern is observed under MNAR, where higher missing rates are also accompanied by lower Kendall’s tau values. For example, in the Laplace-noise setting, Kendall’s tau declines from 0.7827 at 5% missingness to 0.7742 at 20%, because MNAR is driven by unobserved characteristics and higher missingness leads to a greater loss of extreme values, slightly weakening ranking agreement. Overall, the spline smoothing component in the FP-OWA method effectively filters out noise and has little adverse impact on the segmentation and integration steps, yielding stable performance and rankings that remain highly consistent with the true order.

### 4.6 Ranking stability analysis

**Fig 5:**
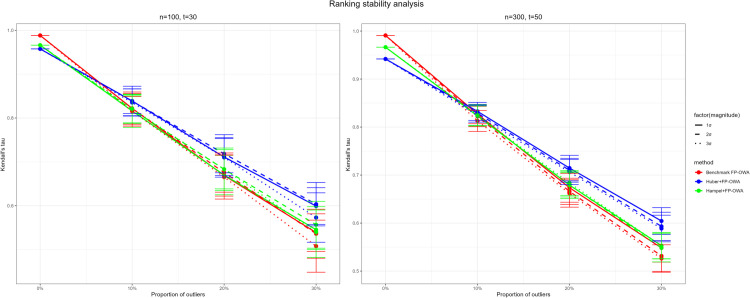
Analysis of ranking stability.

To assess the robustness of the proposed FP-OWA method and its variants for ranking functional data contaminated by outliers, we conduct further simulation studies. We adopt the functional data generation mechanism in Eq 11 and use the true ranking obtained from the integral of the underlying functions as the evaluation benchmark. The proportion of outliers is varied from 0% to 30%, and the perturbation magnitude is set to 1δ, 2δ and 3δ (with *δ* denoting the standard deviation). For each setting, a subset of samples is selected according to the specified proportion and contaminated with Gaussian noise of the corresponding amplitude to mimic data pollution. The performance of three methods—the baseline FP-OWA, the Huber-filtered FP-OWA [35], and the Hampel-filtered FP-OWA [36]—is evaluated using the Kendall’s tau statistic. The simulation results are visualized in Fig 5.

From Fig 5, we can see that in functional data ranking tasks, the FP-OWA method with Huber or Hampel pre-filtering is markedly more robust to outliers than the baseline FP-OWA method. As both the proportion and the magnitude of outliers increase, the ranking stability of all methods declines, but the filtered versions are able to maintain a relatively high level of concordance even under severe contamination. Under the same outlier settings, all methods achieve higher Kendall’s tau values when *n* = 300 and *t* = 50 than when *n* = 100 and *t* = 30. This indicates that a larger sample size dilutes the influence of individual outliers, and a denser time grid captures the overall trend and periodic structure of the functional data more completely, thereby reducing the impact of local anomalies on the integral-based ranking. The FP-OWA variants are also more stable in the large-sample case, as the increased number of time points allows the filters to more accurately identify departures at abnormal time locations, further enhancing ranking stability. We define the method to “break down” when Kendall’s tau falls below 0.7, that is, when ranking stability deteriorates substantially. As shown in the figure, the effective breakdown point of the baseline FP-OWA is relatively low and strongly affected by sample size. When *n* = 100 and *t* = 30, the breakdown occurs when the outlier proportion is around 20%–30%; once this threshold is exceeded, Kendall’s tau quickly drops below 0.7. When *n* = 300 and *t* = 50, the breakdown point improves slightly to around 30%, but it still reflects limited resistance to outliers compared with the variant methods, suggesting that the baseline FP-OWA is fairly sensitive to high proportions or large magnitudes of contamination. By contrast, FP-OWA combined with Huber or Hampel filtering delays the breakdown point by roughly 10%–20% relative to the baseline FP-OWA. The plots further show that the Huber-based variant is more stable at moderate contamination levels, whereas the Hampel-based variant performs better when outliers are more extreme. In summary, when the data contain a high proportion of outliers, applying Huber or Hampel filtering to correct extreme deviations before ranking can effectively mitigate their influence on the integral-based scores. The resulting FP-OWA variants provide a more reliable solution for robust ranking of functional data.

### 4.7 Prediction

**Fig 6:**
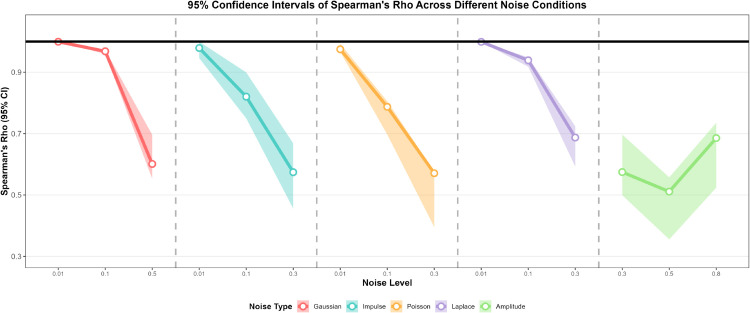
Predictive performance of the FP-OWA method under different noise contaminations.

In this experiment, Monte Carlo simulations are used to systematically evaluate the predictive performance of the FP-OWA method under various noise-contamination scenarios, with the aim of verifying its robustness and stability in ranking complex functional data. The experimental design continues to adopt the functional data generation scheme in Eq 11, to which different types and levels of noise are added in order to mimic realistic contamination settings. We set *n* = 300, *t* = 100, and use Spearman’s rho as the primary metric to quantify the association between the predicted ranking and the true ranking. The experimental results are presented in Fig 6.

The predictive performance of the FP-OWA method is strongly affected by both the type and the level of noise, while its runtime remains stable between 0.07 and 0.10 seconds. This indicates high computational efficiency and makes the method suitable for ranking tasks on moderately sized functional datasets. As shown in Fig 6, FP-OWA performs particularly well at low noise levels. At moderate noise levels, Laplace and Gaussian noise mainly manifest as global fluctuations that can be effectively attenuated by spline smoothing, so FP-OWA adapts well to discrete and heavy-tailed distributions in these settings. By contrast, the extreme spikes or scale shifts induced by impulse and amplitude noise may distort the MBD weights and the segmentation procedure, leading to reduced ranking stability. When the noise level is high, the performance of FP-OWA deteriorates but still remains reasonably robust. Future work could consider incorporating more robust clustering strategies or combining FP-OWA with nonparametric smoothing techniques to further enhance its ability to suppress strong random noise.

## 5 Empirical data analysis

### 5.1 Sources of data

In this section, we use the 24-hour daily average concentrations of PM2.5 and O3 in 13 cities of the Beijing–Tianjin–Hebei region from 1 January 2023 to 31 December 2023 as the objects of study. The data are obtained from the China Air Quality Online Monitoring and Analysis Platform (https://www.aqistudy.cn/historydata/), which provides real-time monitoring information but does not support direct data downloads. Therefore, the dataset used in this paper is taken from the Environmental Research Database of the China Research Data Service(CNRDS) Platform (https://www.cnrds.com/Home/Index#/FinanceDatabase/DB/CEDS/ViewName/

**Fig 7:**
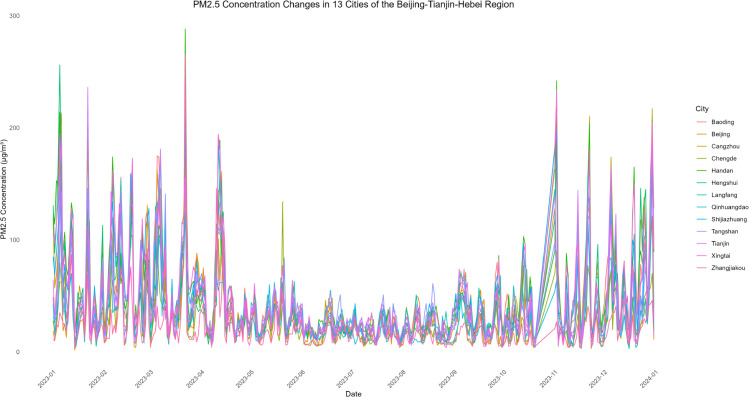
Temporal variation of PM2.5 concentrations in the Beijing–Tianjin–Hebei urban agglomeration.

From Fig 7, it can be seen that the temporal variation of *PM*2.5 concentrations in the Beijing–Tianjin–Hebei urban agglomeration exhibits a pronounced seasonal pattern. The main sources of *PM*2.5 include coal combustion, industrial emissions, fugitive dust, and secondary formation processes, and its concentration is strongly related to meteorological conditions and the regional energy consumption structure. In winter, *PM*2.5 levels rise sharply, with multiple peaks exceeding $200\mu g/m^{3}$. By contrast, summer is the cleanest season, during which the *PM*2.5 concentrations in all 13 cities remain below $50\mu g/m^{3}$ in July. In spring, *PM*2.5 concentrations show intermittently high values and pronounced fluctuations. This is mainly due to frequent dust storms and the recovery of industrial production, which together lead to repeated pollution peaks; however, as precipitation increases and vegetation greens up, concentrations decline in the latter part of the season. In early autumn, meteorological conditions are generally favorable and the dispersion of pollutants is relatively strong. Later in the season, air temperature drops, temperature inversions begin to occur, and potential impacts from regional activities such as straw burning emerge, causing *PM*2.5 levels to gradually converge toward typical winter conditions. Overall, the seasonal pattern can be summarized as “high in winter and spring, low in summer and autumn”.

**Fig 8:**
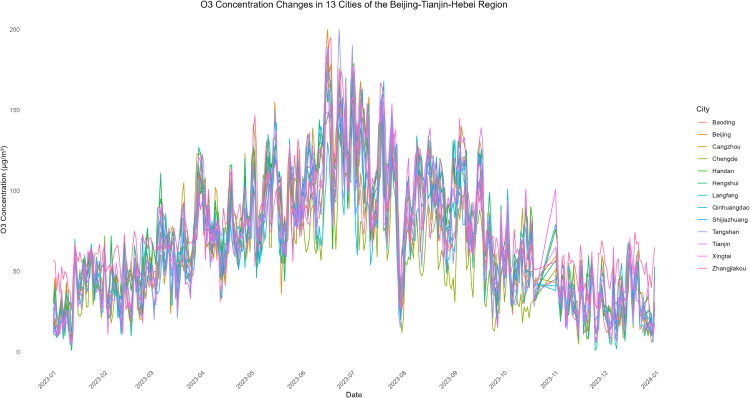
Temporal variation of *O*_3_ concentrations in the Beijing–Tianjin–Hebei urban agglomeration.

*O*_3_ is mainly formed through photochemical reactions between volatile organic compounds (*VOCs*) and nitrogen oxides (*NOx*) under solar radiation, and its concentration is therefore closely linked to air temperature and irradiance. As shown in Fig 8, the *O*_3_ concentration in the Beijing–Tianjin–Hebei urban agglomeration reaches its annual peak in summer; in some cities, daily *O*_3_ levels exceed $180\mu g/m^{3}$ on a number of days in June and July. In contrast, winter is the season with the lowest *O*_3_ concentrations, with most cities remaining below $50\mu g/m^{3}$. During spring, *O*_3_ exhibits a persistent upward trend, increasing from around $50\mu g/m^{3}$ at the beginning of the season to the high-value range in early summer. In autumn, *O*_3_ concentrations gradually decline as temperature drops and solar radiation weakens, falling from high to low levels. Overall, the seasonal pattern can be summarized as “high in summer, low in winter, with spring and autumn as transition periods.” By comparing Figs 7 and 8, it is evident that *PM*2.5 and *O*_3_ in the 13 cities of the Beijing–Tianjin–Hebei region exhibit a pronounced inverse relationship over the annual cycle. This opposite pattern persists across all seasons and clearly reflects the typical pollution differentiation in the region, characterized by “winter haze and summer photochemical ozone.”

**Fig 9:**
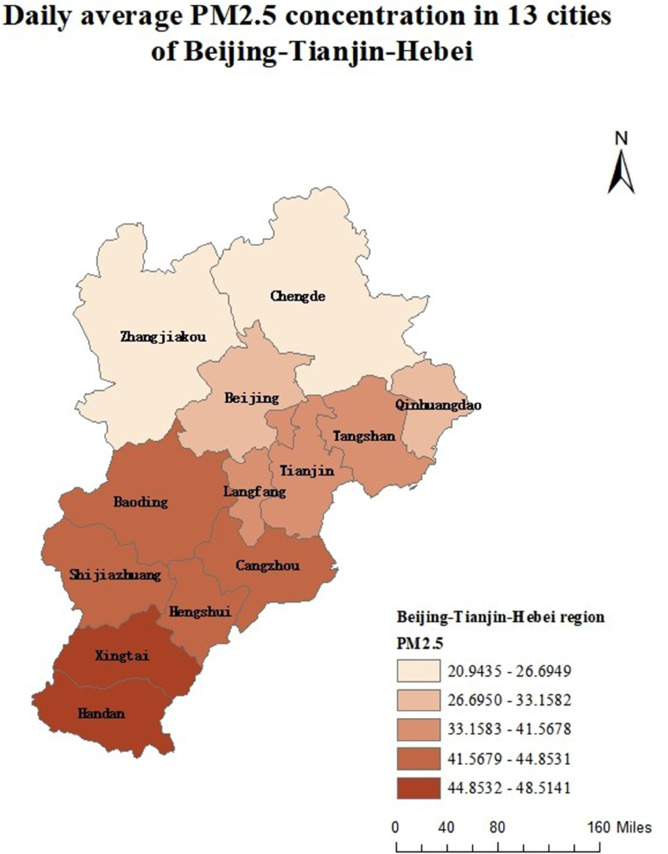
Spatial pattern of average PM2.5 concentrations in the Beijing–Tianjin–Hebei urban agglomeration.

In terms of inter-city differences, the daily mean *PM*2.5 levels clearly follow a south–north gradient, with higher values in the south and lower values in the north. As shown in Fig 9, cities such as Handan and Xingtai fall into the relatively high concentration range, whereas Chengde, Zhangjiakou, and Qinhuangdao exhibit markedly lower average *PM*2.5 levels. Together with Fig 7, it can be seen that even during the pollution season the peak *PM*2.5 concentrations in these northern cities mostly remain below $150\mu g/m^{3}$, indicating comparatively lighter pollution.

**Fig 10:**
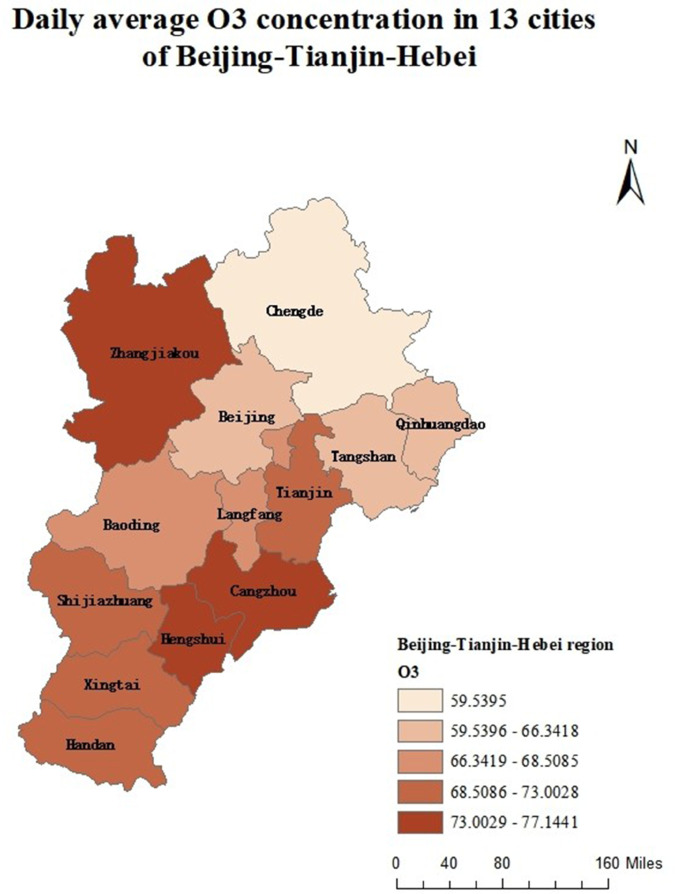
Spatial pattern of average *O*_3_ concentrations in the Beijing–Tianjin–Hebei urban agglomeration.

The daily mean *O*_3_ levels show a “high in inland plain cities, low in coastal cities” configuration. As seen in Fig 10, high values are concentrated in the central and southern plains of Hebei and in areas with intensive industrial activity, with Zhangjiakou, Hengshui, and Cangzhou being representative examples. Under strong solar radiation and high summer temperatures, *O*_3_ formation in these cities is markedly more efficient than elsewhere, and their annual daily means rank among the highest in the region. Low values, by contrast, are mainly found in the northern mountainous areas of Hebei and the eastern coastal zone, typified by Chengde, Qinhuangdao, and Beijing, where *O*_3_ concentrations remain low throughout the year. Overall, the region exhibits a pronounced spatial contrast between *PM*2.5 and *O*_3_ concentration levels.

### 5.2 Application of the FP-OWA method

Using the FP-OWA method, we further analyzed the 2023 daily average concentrations of *PM*2.5 and *O*_3_ for the 13 cities in the Beijing–Tianjin–Hebei region, and the results are as follows.

**Fig 11:**
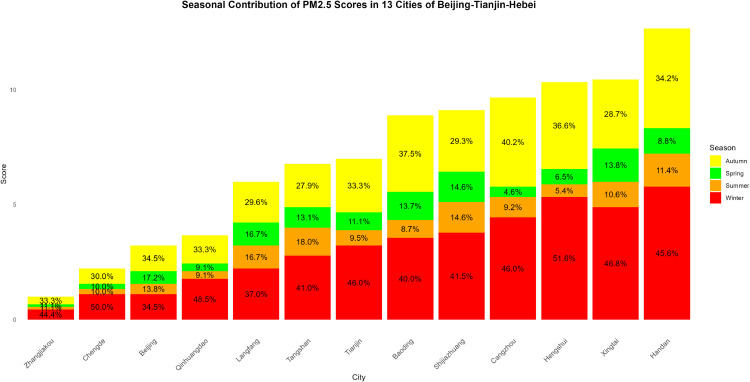
Ranking of PM2.5 concentrations in the Beijing–Tianjin–Hebei urban agglomeration.

Fig 11 shows both the differentiated ranking of *PM*2.5 concentrations among cities and the seasonal contribution pattern for each city. From the perspective of ranking heterogeneity, Handan, Xingtai, and Hengshui occupy the top positions in terms of *PM*2.5 levels within the urban agglomeration. This is mainly because these three cities are traditional high–energy-consumption, high-emission centres, with large industrial and agricultural emission bases. Combined with stagnant winds and temperature inversions in the hinterland of the North China Plain, pollutants tend to accumulate. In addition, these cities lie at key nodes of the *PM*2.5 transport corridor in the Beijing–Tianjin–Hebei region, so inflow from upwind areas further elevates local concentrations, jointly supporting their high rankings. By contrast, Zhangjiakou, Chengde, and Beijing fall into the lower quantiles of the regional *PM*2.5 distribution. Zhangjiakou and Chengde benefit from the mountainous terrain in northern Hebei, which enhances vertical dispersion, and their economies are dominated by eco-tourism and light industries with relatively low emission intensity. Although Beijing is a large metropolis, long-term stringent air-pollution control has substantially reduced local emissions, and the city is frequently influenced by cleaner air masses from the north, leading to weaker contributions from regional transport. These factors together result in the relatively low ranking of *PM*2.5 concentrations in these cities.

**Fig 12:**
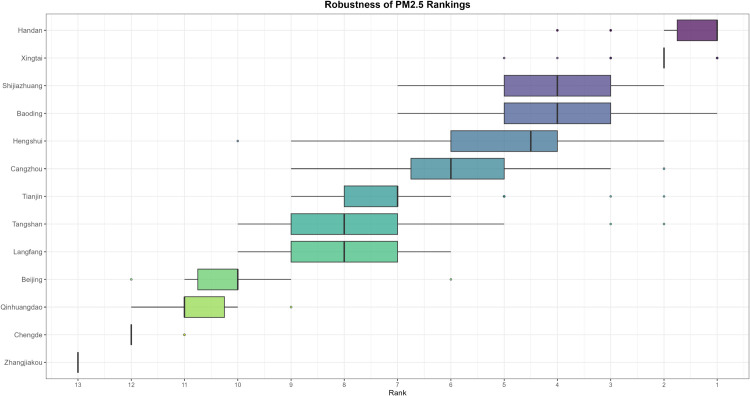
Robustness analysis of PM2.5 rankings.

To assess the reliability of the above ranking results, we adopt a bootstrap procedure. Specifically, we first extract the residuals from the smoothed functional data and perform 200 random resamples with replacement, which are then added back to the fitted signals to generate bootstrap datasets contaminated with random noise. For each bootstrap sample, the FP-OWA algorithm is rerun to obtain a new ranking. The distribution of the 200 bootstrap rankings is finally summarized by boxplots, as shown in Fig 12, providing a visual representation of the uncertainty range associated with the ranking results.

As seen in Fig 12, the overall structure exhibits a clear diagonal distribution, which demonstrates that the FP-OWA method has strong discriminative power and effectively captures the structural differences in the data rather than random noise. Specifically, the box plots for the cities at the extremes of the ranking—Handan (ranked first) and Zhangjiakou (ranked last)—are relatively narrow, indicating good stability. However, for cities in the middle range, such as Tianjin and Tangshan, the boxes are wider and overlap, which reflects the homogenized competition in *PM*2.5 pollution control levels in the central part of the Beijing–Tianjin–Hebei region. This suggests that the performance differences among these cities are not statistically significant.

**Fig 13:**
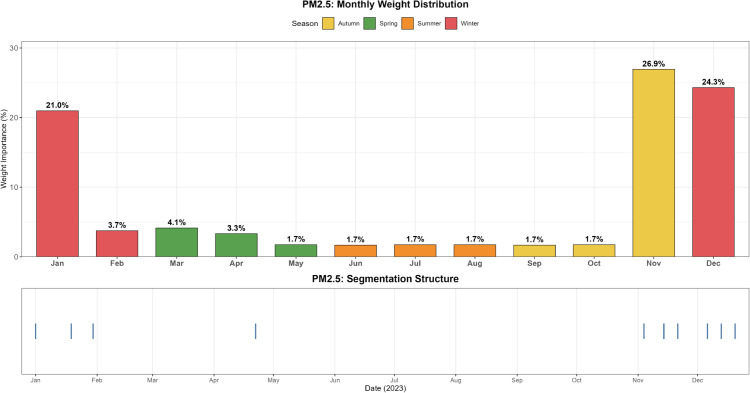
Monthly contribution weights and temporal segmentation of PM2.5 in the Beijing–Tianjin–Hebei urban agglomeration.

Fig 13 summarizes the seasonal–monthly contribution patterns of *PM*2.5 in the Beijing–Tianjin–Hebei region and the segmentation structure obtained with the FP-OWA method. From the perspective of contribution weights, the MBD-based weights exhibit a clear U-shaped pattern: December and January are the months with the highest pollution contribution, with a monthly share typically exceeding 20%, far above the other months. This highlights a “winter–autumn dominated” pattern of *PM*2.5 pollution in the region. From the viewpoint of temporal segmentation, the DBSCAN clustering algorithm automatically divides the year into four regimes: a winter accumulation period, a spring dust-fluctuation period, a summer low-pollution stable period, and an autumn transition period. The segment boundaries align closely with key turning points in the *PM*2.5 concentration series, such as the onset of coal-fired heating in winter, the increase in precipitation in summer, and the emergence of temperature inversions in autumn. This not only captures the common features of regional pollution, but also confirms the rationality and adaptability of the automatic segmentation strategy embedded in the FP-OWA method. Combining Fig 11 with the seasonal contribution shares for each city reveals substantial differences in both the strength of seasonal dominance and the underlying driving mechanisms. For example, Handan, as a national core base of steel production capacity, has a relatively high share of annual primary particulate emissions from sintering and blast-furnace ironmaking during winter. Combined with low-level emissions from residential coal heating, this forms a dual driving mechanism of industrial and coal-combustion sources. Moreover, steel production in autumn does not undergo seasonal shutdown, so particulate emissions persist, and the high frequency of stagnant winds weakens dispersion, leading to a secondary peak in contribution. In Hengshui, the winter share of *PM*2.5 emissions is the highest in the region, reaching 51.6%. The city lies in the central plain, where winter stagnation is frequent and pollutants tend to be trapped in local recirculation. As a major agricultural city, autumn straw burning combined with chemical emissions pushes the autumn share up to 36.6%. In Beijing, by contrast, the winter and autumn shares of *PM*2.5 emissions are perfectly balanced at 34.5% each. Residential coal use has essentially been eliminated, winter emissions are dominated by traffic, and regional joint prevention and control measures help curb cross-boundary transport. There is no straw burning in autumn, and the main pollution source is inflow from surrounding areas, leading to the roughly symmetric winter–autumn contributions. Increased precipitation and dust retention by vegetation further reduce concentrations in spring. For Chengde and Zhangjiakou, the winter share is relatively high (50% and 44.4%, respectively), but the absolute concentration level is only about one third to one half of that in the high-pollution cities. Both cities are dominated by eco-tourism and light industry, with generally low industrial emission intensity. Although coal-fired heating is still used in winter, it is largely replaced by clean energy, and the mountainous terrain brings frequent cold-air activities and strong vertical dispersion of particulates. As a result, the winter contribution share is high, but the actual *PM*2.5 concentration remains relatively low.

**Fig 14:**
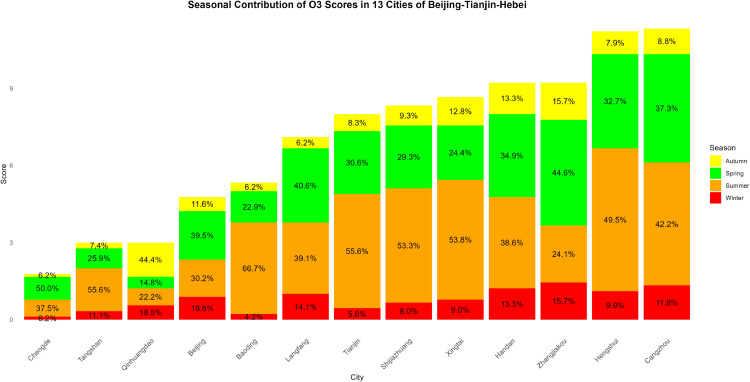
Ranking of *O*_3_ concentrations in the Beijing–Tianjin–Hebei urban agglomeration.

Unlike the spatial pattern of *PM*2.5, the ranking of *O*_3_ concentrations in Fig 14 reveals two distinct clusters. Cangzhou, Hengshui, and Zhangjiakou form a high-*O*_3_ cluster, mainly distributed across the central–southern plains and the transitional zone to northern Hebei. Chengde, Tangshan, and Qinhuangdao constitute a low-*O*_3_ cluster, covering the northern mountainous areas, heavy-industry cities, and coastal zones. The key advantage of the high-value cluster lies in the ample supply of precursors and favorable photochemical conditions. For example, Cangzhou and Hengshui are major centres of chemical and agricultural industries, with large baseline emissions of *VOCs* and *NOx*; Zhangjiakou, although relatively weak in industrial emissions, experiences an early onset of photochemical activity in spring, and its local topography is conducive to *O*_3_ accumulation. Together, these factors support their high rankings. By contrast, Chengde benefits from strong dispersion associated with its mountainous terrain and low precursor emissions, so *O*_3_ is more easily diluted. Tangshan, despite strong photochemical activity in summer, has high *NOx* emissions that trigger the “*NOx* titration effect”, which consumes *O*_3_. Qinhuangdao is influenced by a maritime climate, where lower temperatures and higher humidity suppress photochemical reactions. These factors jointly lead to relatively low *O*_3_ concentrations in the low-value cluster.

**Fig 15:**
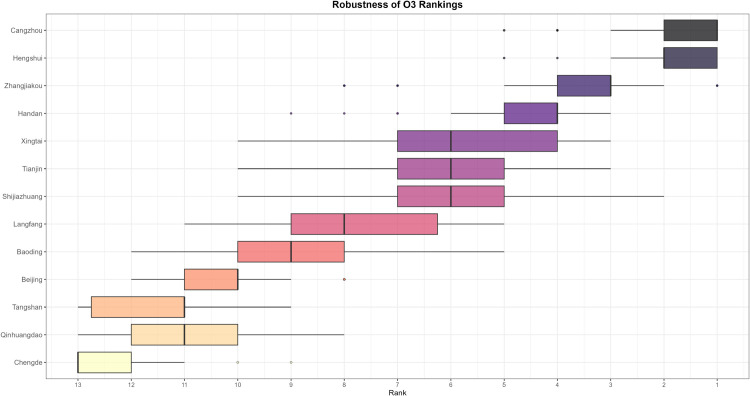
Robustness analysis of *O*_3_ rankings.

Fig 15 displays the stability of *O*_3_ rankings based on 200 bootstrap resamplings, using boxplots. From the figure, it can be seen that the overall ranking follows a clear stepped distribution, demonstrating that the FP-OWA method has strong discriminative power. Cangzhou, ranked at the top, and Chengde, ranked at the bottom, exhibit narrow confidence intervals, indicating that their rankings are statistically stable and not affected by data fluctuations. Cities in the middle of the ranking, such as Tianjin and Shijiazhuang, show considerable overlap in their boxplots, with wider spans. This objectively reflects the high homogeneity in *O*_3_ governance levels among central cities in the Beijing–Tianjin–Hebei region, where competition is intense and performance differences are not statistically significant.

**Fig 16:**
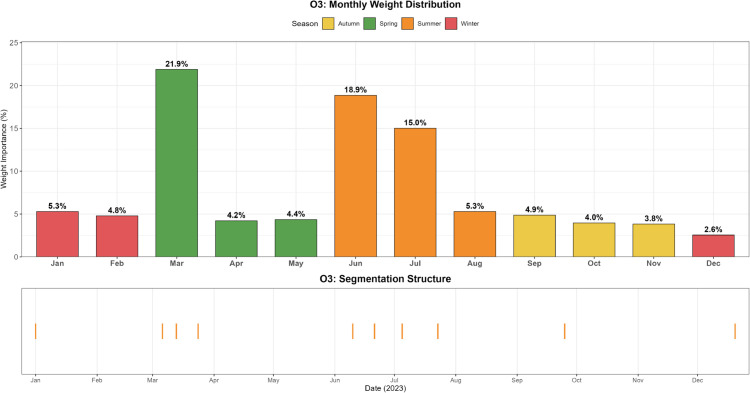
Monthly contribution weights and temporal segmentation of *O*_3_ in the Beijing–Tianjin–Hebei urban agglomeration.

From the perspective of seasonal and monthly contribution patterns in Fig 16, the *O*_3_ weights obtained by the MBD method exhibit an inverted U-shaped profile that is almost the opposite of that for *PM*2.5. March shows the largest contribution, accounting for about 21.9% of the annual total, and marks the transition from winter to spring in the Beijing–Tianjin–Hebei region, when the solar elevation angle rises rapidly and daylight hours increase markedly. June and July are the core months for *O*_3_ pollution, with monthly weights as high as 15%–19%, indicating a pronounced seasonal concentration, while the contributions of the remaining months are relatively small, generally below 7%. The segmentation in Fig 16 is obtained automatically based on local data density, clearly distinguishing a summer photochemical peak period, a spring precursor accumulation period, a winter low-activity trough, and an autumn diffusion–transition period. These segments align closely with the key drivers of *O*_3_ formation, providing further evidence for the effectiveness of the dynamic segmentation strategy in the FP-OWA method. Because *O*_3_ formation depends on precursor supply and photochemical energy, combining Fig 14 with the seasonal contribution shares of each city suggests that the cities in the region can be grouped into four types: summer-dominated, spring–summer co-dominated, spring-dominated, and autumn-special-dominated. For summer-dominated cities, the defining feature is that summer *O*_3_ accounts for more than 50% of the annual total, significantly higher than in other seasons, with Xingtai and Shijiazhuang as typical examples. Xingtai is a mixed industrial–agricultural city in southern Hebei, where *VOCs* emissions from chemical industrial parks are high in summer; combined with high temperatures and strong solar radiation, photochemical reaction rates are markedly higher than in spring. Shijiazhuang, the provincial capital, experiences the superposition of traffic *NOx* emissions and *VOCs* emissions from surrounding chemical industries. Located on the eastern foothills of the Taihang Mountains, the city is affected by föhn-like warming in summer, which further enhances photochemical activity, making summer the season with the largest contribution share. The spring–summer co-dominated type is characterized by a combined spring-plus-summer share exceeding 70%, indicating strong seasonal continuity. Cangzhou, a major base for petroleum and chlor-alkali industries, shows this pattern: springtime resumption of production boosts *VOCs* and *NOx* emissions, while in summer, high temperatures and strong radiation further increase *VOCs* emissions from chemical parks, and the high frequency of stagnant conditions over the plains favors *O*_3_ accumulation; together, spring and summer account for 79.5% of the total. Hengshui, with its distinctive rubber, fiberglass, and agricultural sectors, also falls into this category. In spring, *VOCs* emissions from rubber vulcanization and *NOx* emissions from fertilizer application provide abundant precursors; in summer, industrial parks release large amounts of *VOCs* that efficiently react with traffic-related *NOx*, and the spring–summer share reaches 82.2%. Spring-dominated cities are those where the spring *O*_3_ share exceeds that of summer. Zhangjiakou is a representative high-plateau city in northern Hebei. Rapid warming in spring triggers photochemical activity earlier than in surrounding areas, and the relatively enclosed topography of the Bashang Plateau leads to a high frequency of stagnant conditions, making local *O*_3_ accumulation easier. Although summer temperatures are higher, evapotranspiration from grassland vegetation increases humidity and suppresses photochemical reactions, so the spring dominance is pronounced. Chengde, an ecological city in the northern mountains, also belongs to this type. Strong springtime insolation and photochemical activity are offset by a local economy dominated by eco-tourism and light industry, resulting in limited *VOCs* and *NOx* precursor emissions. In summer, more frequent rainfall enhances wet deposition and removes precursors, while strong vertical mixing in mountainous terrain promotes dispersion. Consequently, Chengde has the highest spring share among all cities in the region, but the lowest absolute *O*_3_ concentration. The autumn-special-dominated type is characterized by a markedly higher autumn share than in other seasons, and within the Beijing–Tianjin–Hebei urban agglomeration there is only one such city: Qinhuangdao. As a coastal city, Qinhuangdao experiences strong marine humidity in summer, which suppresses photochemical reactions and leads to lower *O*_3_ levels than in inland areas. In autumn, before frequent intrusions of cold air from the north, wind speeds decrease relative to summer, slowing *O*_3_ dispersion. At the same time, autumn is the tourism off-season, so traffic *NOx* emissions decline, while industrial *VOCs* transported from Tangshan and Tianjin still provide a supply of precursors under mild photochemical conditions. As a result, autumn accounts for 44.4% of the annual *O*_3_ contribution, making Qinhuangdao the only city where autumn is the dominant season. However, due to generally low photochemical efficiency throughout the year, its overall *O*_3_ level ranks only 11th among the 13 cities.

In summary, the ranking differentiation and seasonal contribution patterns of *PM*2.5 and *O*_3_ concentrations in the Beijing–Tianjin–Hebei urban agglomeration exhibit pronounced spatial gradients and seasonal regularities. These findings provide a systematic basis for addressing the dual pollution patterns of *PM*2.5—characterized by “higher in the south, lower in the north, dominated by winter and autumn”—and *O*_3_—characterized by “higher on the plains, lower in coastal areas, dominated by summer and spring”. To improve regional air quality in an integrated manner, priority should be given to establishing a joint prevention and control mechanism that operates across administrative boundaries and to advancing comprehensive management of pollution sources, thereby accelerating the formation of a low-carbon regional development model. Concretely, this requires the creation of a unified environmental standards system, the improvement of ecological compensation schemes, and the promotion of green industrial transformation, so as to achieve coordinated improvement and balanced development of environmental quality across the region.

## 6 Conclusion

The FP-OWA method proposed in this study offers a new perspective for ranking complex functional data. By integrating spline smoothing, depth-based analysis, and rank statistics, it effectively improves both the accuracy and stability of the ranking results. In terms of methodological performance, the simulation study systematically evaluates FP-OWA under five types of noise, three missing-data mechanisms, and outlier proportions ranging from 0% to 30%. Under low-noise conditions, FP-OWA clearly outperforms FPCA, which suffers from low rank concordance, the computationally intensive WLR method, and the depth-based h-MD and RTD methods, which exhibit systematic bias. Even in high-noise settings or under severe missingness, FP-OWA still maintains a noticeable advantage. Moreover, when combined with Huber or Hampel pre-filtering, the breakdown point with respect to outliers is delayed by about 10%–20%, providing strong evidence of the method’s stability in the presence of complex data perturbations. From an applied perspective, employing FP-OWA to rank the daily mean *PM*2.5 and *O*_3_ concentrations in the Beijing–Tianjin–Hebei urban agglomeration allows us to accurately uncover the spatio-temporal heterogeneity of regional air pollution, thereby supplying robust technical support and data evidence for environmental governance and policy-making.

Although the FP-OWA method exhibits excellent computational efficiency at the sample sizes considered in the simulation study, the DBSCAN clustering and MBD depth modules embedded in the framework still face potential computational and theoretical challenges when dealing with ultra–large-scale or high-dimensional functional data. First, there is the scalability issue of MBD depth computation. Although MBD is simpler than traditional bandwidth-based depth measures, its core logic still relies on pairwise comparisons or higher-order combinations of sample curves, so the time complexity is essentially on the order of *O*(*N*^2^). As the sample size ngrows to massive scales, this quadratic increase in computational cost will lead to a substantial rise in runtime. In addition, when the sampling frequency of high-dimensional curves (dimension *T*) is very high, the cost of each integral comparison accumulates, which restricts the responsiveness of the algorithm in real-time or high-frequency streaming data applications. Second, there is the question of how suitable DBSCAN is in high-dimensional feature spaces. In this paper, DBSCAN is used for feature partitioning and relies on distance measures between samples (such as Euclidean distance) to define density. However, when facing high-dimensional functional data or inflated high-dimensional representations, the method inevitably encounters the curse of dimensionality. In high-dimensional space, data distributions become sparse and the differences between pairwise distances are blurred, making density-based cluster structures difficult to identify accurately. At the same time, in the absence of efficient spatial indexing structures, the neighborhood search process of DBSCAN also requires an *O*(*N*^2^)-scale distance matrix, which further constrains the applicability of FP-OWA in ultra–high-dimensional, complex data settings.

Given both the promise demonstrated by FP-OWA and the challenges it faces in handling ultra–large or high-dimensional data, future work can proceed along at least three directions. First, to address the quadratic growth in the cost of MBD depth computation, it would be useful to develop fast approximate depth algorithms that substantially reduce computational complexity while preserving ranking accuracy, and to design parallel MBD algorithms within a distributed computing framework to enable efficient processing of massive functional datasets. Second, to alleviate DBSCAN’s failure in high-dimensional feature spaces due to the curse of dimensionality, future research may explore manifold learning techniques as alternatives to traditional Euclidean distance, so as to capture more precisely the intrinsic nonlinear geometry of functional data in high-dimensional spaces. Third, since the current FP-OWA framework is mainly designed for univariate functional data, subsequent work will aim to extend it to multivariate functional settings by constructing multivariate feature aggregation operators that simultaneously capture cross-variable correlations and spatio-temporal dependencies, thereby further validating and enhancing the practical utility of the method.

## Supporting information

S1 Appendix Proof process of asymptotic properties. (PDF)

S2 Code The complete simulation workflow involved in this study has been implemented in R (version 4.3.0). The simulation source code (FP-OWA.R) has been deposited in a GitHub repository (https://github.com/Ly-sxmb/FP-OWA). The code structure comprises functional data generation, noise addition, implementation of five comparative methods, and parallelized performance evaluation, all of which correspond directly to the methodological procedures described in the manuscript.
(R)

S3 Raw Data The raw data used in the empirical study have been deposited in a GitHub repository (https://github.com/Ly-sxmb/FP-OWA). (CSV)
