# Supplementary material for: The adaptive functional piecewise ordered weighted averaging method and its application to pollutant concentration analysis
Source: PLoS One. 2026 Feb 13;21(2):e0342192. doi: 10.1371/journal.pone.0342192 (PMC12904593; doi:10.1371/journal.pone.0342192)
Supplement: S1 Appendix — (PDF) [file pone.0342192.s001.pdf]

Appendix

Theorem 1 Proof: First, the function $f_i(t)$ is expressed by expanding it in terms of spline basis functions:

$$f_i(t) = \sum_{k=1}^{K} \beta_{ik}\phi_k(t),$$

where $\phi_k(t)$ represents the spline basis functions, β_{ik} denotes the coefficients to be estimated, and K is the number of basis functions. The coefficients β_i are estimated by minimizing the regularized objective function:

$$\min_{\beta_i} \left\{ \sum_{j=1}^{n} [y_i(t_j) - \sum_{k=1}^{K} \beta_{ik}\phi_k(t_j)]^2 + \lambda \int_0^T [f_i''(t)]^2 dt \right\}.$$

The first term represents the fitting error, while the second term serves as a penalty to control the smoothness of the estimation. $\lambda > 0$ denotes the penalty parameter. The penalty term can be expanded as follows:

$$\int_0^T [f_i''(t)]^2 dt = \int_0^T [\sum_{k=1}^{K} \beta_{ik}\phi_k''(t)]^2 dt = \beta_i^\top \Omega \beta_i.$$

Matrix Ω represents the inner product matrix of the second derivatives of the basis functions, with elements $\Omega_{kl} = \int_0^T \phi_k''(t)\phi_l''(t)dt$. Let matrix Φ denote the design matrix of the basis functions at the observation points, where the elements satisfy $\Phi_{jk} = \phi_k(t_j)$, $Y_i = (y_i(t_1), ..., y_i(t_n))^\top$. The objective function can then be expressed in matrix form as:

$$\min_{\beta_i} (Y_i - \Phi\beta_i)^\top (Y_i - \Phi\beta_i) + \lambda \beta_i^\top \Omega \beta_i.$$

By differentiating the objective function and setting the result to zero, the explicit solution for the estimated coefficients is obtained:

$$\hat{\beta}_i = (\Phi^\top \Phi + \lambda\Omega)^{-1} \Phi^\top Y_i.$$

According to Wahba G.(1990), when conditions $\lambda \to 0$ and $n\lambda \to \infty$ are met, the mean squared error of the estimated function satisfies:

$$E\left[\left\| \hat{f}_i - f_i \right\|_2^2 \right] = E\left[\int_0^T (\hat{f}_i(t) - f_i(t))^2 dt \right] = O\left((n^{-1})^{2r/(2r+1)} \right),$$

where r is the smoothness parameter of the function f_i, indicating the order of continuity of its derivatives. Furthermore, Stone(1982) established the uniform consistency of the smoothing spline estimator under suitable conditions:

$$\left\| \hat{f}_i - f_i \right\|_\infty = \sup_{t \in [0,T]} \left| \hat{f}_i(t) - f_i(t) \right| = O_p\left(\left(n^{-1} \log n \right)^{2r/(2r-1)} \right).$$

The Generalized Cross-Validation (GCV) method should be considered for the automatic selection of the penalty parameter λ.

$$GCV(\lambda) = \frac{n^{-1} \parallel Y_i - \hat{Y}_i(\lambda) \parallel^2}{[n^{-1} tr(I - A(\lambda))]^2},$$

where $\hat{Y}_i(\lambda) = \Phi\hat{\beta}_i(\lambda)$ denotes the fitted values, and $A(\lambda) = \Phi(\Phi^\top\Phi + \lambda\Omega)^{-1}\Phi^\top$ denotes the smoothing matrix. According to Craven and Wahba(1978), the λ selected by GCV satisfies $\lambda_{GCV} \approx n^{-2r/(2r+1)}$, which implies that when $n \to \infty$ holds, both $\lambda_{GCV} \to 0$ and $n\lambda_{GCV} \to \infty$ are true. Therefore, when $n \to \infty$ is met, for any $\varepsilon > 0$, the following holds:

$$P\left(\sup_{t\in[0,T]} \mid \hat{f}_i(t) - f_i(t) \mid > \varepsilon\right) \leq \frac{E[\left\|\hat{f}_i - f_i\right\|_\infty]}{\varepsilon} \to 0.$$

That is, the estimator function $\hat{f}_i(t)$ converges in probability to the true function $f_i(t)$.

Therefore, by combining Chebyshev's inequality with the above convergence rate, when $n \to \infty$ holds, for any $\varepsilon > 0$:

$$P\left(\sup_{t\in[0,T]} \mid \hat{f}_i(t) - f_i(t) \mid > \epsilon\right) \leq \frac{C}{\varepsilon^2} \cdot \left(\frac{\log n}{n}\right)^{2r/(2r+1)} \to 0,$$

where C is a constant related to the smoothness of the function and the level of noise. This completes the proof of Theorem 1.

Theorem 2 Proof: The fundamental principle of the DBSCAN algorithm is density-based clustering. For any data point x, its $\epsilon-$neighborhood is defined as $N_\epsilon(x) = \{y \in X : \zeta(x,y) \leq \epsilon\}$, where $\zeta(x,y)$ denotes the distance metric and X represents the dataset. The core point, border point, and noise point are defined as follows:

(1) Core point: If $\mid N_\epsilon(x) \mid \geq minPts$, then x is considered a core point;

(2) Border point: If $\mid N_\epsilon(x) \mid < minPts$, but x lies within the ϵ-neighborhood of a core point;

(3) Noise point: A point that is neither a core point nor a border point.

Near the segmentation points of functional data, data characteristics (such as mean, variance, or autocorrelation) undergo significant changes, leading to differences in the density distribution of the standardized data $z_i(t)$ across different segments. Specifically, let $t_j \in T$ be the true segmentation point; then, a density change occurs in the vicinity of t_j:

$$\lim_{\Delta t\to 0} \frac{P(z \in N_\epsilon(t_j - \Delta t))}{P(z \in N_\epsilon(t_j + \Delta t))} \neq 1.$$

Based on the DBSCAN algorithm proposed by Ester et al.(1996), the method is capable of identifying boundaries between regions with varying densities when such variations are present in the data. In particular, near segmentation points, where density changes occur, data points are often assigned to different clusters or identified as noise points. Theoretical results by Sander et al.(1998) demonstrate that, with appropriate parameter selection, the clustering results of DBSCAN asymptotically converge to the true density level sets. Specifically, for any $\delta > 0$, there exists a sample size n such that for all $n > n_0$:

$$P\left(\max_{1\leq j\leq \hat{m}} \min_{1\leq k\leq m} \mid t_j - \hat{t}_k \mid < \delta \text{ and } \max_{1\leq k\leq m} \min_{1\leq j\leq \hat{m}} \mid t_j - \hat{t}_k \mid < \delta\right) > 1 - \alpha.$$

The above inequality is equivalent to:

$$P(\mathrm{H}(T, \hat{T}_n) < \delta) \to 1 - \alpha,$$

where the Hausdorff distance is defined as

$$H\left(A,B\right) = \max\left\{\sup_{a\in A} \inf_{b\in B} \zeta(a,b), \sup_{b\in B} \inf_{a\in A} \zeta(a,b)\right\}.$$

When parameter ϵ varies with the sample size n and satisfies conditions $\epsilon \to 0$ and $n\epsilon^{\zeta} \to \infty$, the clustering result of DBSCAN asymptotically converges to the true density level set. In the proposed method, the ϵ parameter—automatically determined using the k-distance graph and the elbow method—meets these conditions. Specifically, the k-distance graph is generated by sorting the distances from each point to its k-th nearest neighbor in ascending order. The elbow method then selects the "knee point" of the k-distance graph as the ϵ value. When the dataset is sufficiently large, this approach can adaptively detect abrupt changes in data density, thereby ensuring the consistency of the segmentation results. Moreover, when minPts is set as a multiple of the data dimensionality, together with the adaptively selected ϵ parameter:

$$\lim_{n\to\infty} P(\mathrm{H}(T, \hat{T}_n) < \delta) = 1.$$

Therefore, when the sample size is sufficiently large, the DBSCAN-based automatic segmentation method is guaranteed to converge to the true segmentation points with probability 1.

Theorem 3 Proof: The band depth of the function is defined as follows according to López-Pintado and Romo(2009):

$$MBD(f) = E\left[\psi\left\{t \in [0,T] : \min\left(X_1(t), X_2(t)\right) \le f(t) \le \max\left(X_1(t), X_2(t)\right)\right\}\right],$$

where X_1, X_2 refers to a pair of random functions independently drawn from the population distribution, and ψ denotes the Lebesgue measure. Intuitively, the $MBD(f)$ represents the average proportion of time that the function f is covered by the "band" formed by two randomly selected functions. A higher depth value indicates that the function is more "central" and better captures the central tendency of the population distribution. The sample band depth is defined as follows:

$$MBD_n(f) = \frac{1}{n(n-1)/2} \sum_{i<j} \psi\left\{t \in [0,T] : \min\left(f_i(t), f_j(t)\right) \le f(t) \le \max\left(f_i(t), f_j(t)\right)\right\},$$

where f_i and f_j are functions within the sample. For each segment $[t_j, t_{j+1}]$, the MBD values are normalized as follows:

$$MBD_n^j(f) = \frac{1}{n(n-1)/2} \sum_{i<k} \psi\{t \in [t_j, t_{j+1}] \colon \min(f_i(t), f_k(t)) \le f(t) \le \max(f_i(t), f_k(t))\} / \left|t_{j+1} - t_j\right|.$$

Definition of function

$$g_{i,j}(f) = \psi t \in [0,T] : \min(f_i(t), f_j(t)) \le f(t) \le \max(f_i(t), f_j(t)),$$

then

$$MBD_n(f) = \frac{1}{n(n-1)/2} \sum_{i<j} g_{i,j}(f).$$

According to the law of large numbers, when $n \to \infty$ occurs,

$$MBD_n(f) \xrightarrow{a.s.} E[g_{1,2}(f)] = MBD(f).$$

To derive the uniform convergence rate, concentration inequalities for U-statistics must be employed. Based on the U-process theory of Arcones and Giné(1993), for a bounded and measurable function class $\mathcal{F}$, there exists a constant $C > 0$ such that for any $\epsilon > 0$,

$$P\left(\sup_{f\in\mathcal{F}} \mid MBD_n(f) - MBD(f) \mid > \varepsilon\right) \le 2\exp\left(-2n\varepsilon^2/C\right).$$

Define $A_n = \left\{\sup_{f\in\mathcal{F}} \mid MBD_n(f) - MBD(f) \mid > \varepsilon\right\}$, then

$$\sum_{n=1}^{\infty} P(A_n) \leq \sum_{n=1}^{\infty} 2\exp(-2n\varepsilon^2/C) = 2\sum_{n=1}^{\infty} e^{-kn}.$$

Since the geometric series $\sum_{n=1}^{\infty} e^{-kn}$ converges, the Borel-Cantelli lemma can be applied: if $\sum_{n=1}^{\infty} P(A_n) < \infty$ holds, then $P(\lim_{n\to\infty} \sup A_n) = 0$ follows. In other words, $P(\sup_{f\in\mathcal{F}} |MBD_n(f) - MBD(f)| > \epsilon \text{ i.o.}) = 0$. This means that for any $\epsilon > 0$:

$$P\left(\lim_{n\to\infty} \sup_{f\in\mathcal{F}} \mid MBD_n(f) - MBD(f)| = 0\right) = 1,$$

then

$$\sup_{f\in\mathcal{F}} \mid MBD_n(f) - MBD(f) \mid \xrightarrow{a.s.} 0.$$

This indicates that the sample band depth is a consistent and convergent estimator of the population band depth. Since the sample MBD values within each segment converge to their corresponding population MBD values, the segment weights $w_j = MBD_n^j / \sum_{k=1}^{m} MBD_n^k$ also converge to the corresponding population weights by the continuous mapping theorem. Specifically, when $n \to \infty$,

$$\hat{w}_j = \frac{MBD_n^j}{\sum_{k=1}^{m} MBD_n^k} \xrightarrow{a.s.} \frac{MBD^j}{\sum_{k=1}^{m} MBD^k} = w_j.$$

Theorem 4 Proof: First, by recalling the result from Theorem 1, it is established that the estimator function $\hat{f}_i(t)$ converges uniformly to the true function $f_i(t)$:

$$\sup_{t\in[0,T]} \mid \hat{f}_i(t) - f_i(t) \mid \xrightarrow{p} 0.$$

For the j-th subinterval $[t_j, t_{j+1}]$, the integral of the function $f_i(t)$ is denoted by $I_j(f_i) = \int_{t_j}^{t_{j+1}} f_i(t)dt$, and the corresponding estimated integral is $\hat{I}_j(f_i) = \int_{t_j}^{t_{j+1}} \hat{f}_i(t)dt$. Therefore, we have

$$\left|\hat{I}_j(f_i) - I_j(f_i)\right| = \left|\int_{t_j}^{t_{j+1}} \left[\hat{f}_i(t) - f_i(t)\right] dt\right| \leq \int_{t_j}^{t_{j+1}} \left|\hat{f}_i(t) - f_i(t)\right| dt.$$

Application of the property of uniform convergence

$$\left|\hat{I}_j(f_i) - I_j(f_i)\right| \leq \int_{t_j}^{t_{j+1}} \left|\hat{f}_i(t) - f_i(t)\right| dt \leq (t_{j+1} - t_j) \cdot \sup_{t\in[t_j,t_{j+1}]} \left|\hat{f}_i(t) - f_i(t)\right|.$$

From the result of Theorem 1: when $n \to \infty$ holds, $\sup_{t\in[t_j,t_{j+1}]} \mid \hat{f}_i(t) - f_i(t) \mid \xrightarrow{p} 0$, therefore

$$|\hat{I}_j(f_i) - I_j(f_i)| \xrightarrow{p} 0.$$

It is shown that for each function f_i and each segment j, the estimated integral value converges to the true integral value. For segment j, the true rank $R_j(f_i)$ is defined.

$$R_j(f_i) = 1 + \sum_{k=1,k\neq i}^{n} \mathbb{I}(I_j(f_k) < I_j(f_i)),$$

where $\mathbb{I}(\cdot)$ denotes the indicator function. Similarly, the estimated rank is defined as:

$$\hat{R}_j(f_i) = 1 + \sum_{k=1,k\neq i}^{n} \mathbb{I}(\hat{I}_j(f_k) < \hat{I}_j(f_i)).$$

For any pair of functions (f_i, f_k), if $I_j(f_i) \neq I_j(f_k)$, then there exists a $\gamma_{ik} > 0$ such that

$$\mid I_j(f_i) - I_j(f_k) \mid \geq \gamma_{ik}.$$

Define $\gamma = \min_{i \neq k} \gamma_{ik}$, and then $\gamma > 0$ holds (assuming no parallel cases). When $\mid \hat{I}_j(f_i) - I_j(f_i) \mid < \gamma/2$ holds for all i, the relative order of the estimated integral values matches the relative order of the true integral values.

For any $\epsilon > 0$, when n is sufficiently large, $P(|\hat{I}_j(f_i) - I_j(f_i)| < \gamma/2) > 1 - \epsilon$. For all i and k, it holds that

$$\mathbb{I}(\hat{I}_j(f_k) < \hat{I}_j(f_i)) = \mathbb{I}(I_j(f_k) < I_j(f_i)),$$

thereby

$$\hat{R}_j(f_i) = R_j(f_i),$$

therefore

$$P(\hat{R}_j(f_i) = R_j(f_i)) \geq P(|\hat{I}_j(f_i) - I_j(f_i)| < \gamma/2) > 1 - \epsilon,$$

when $n \to \infty$ occurs, ϵ can become arbitrarily small, therefore,

$$\lim_{n \to \infty} P\left(\hat{R}_j(f_i) = R_j(f_i)\right) = 1.$$

This indicates that, when the sample size is sufficiently large, the rank determined from the estimated function is almost certainly equal to the rank determined from the true function.

Theorem 5 Proof: Consider Theorem 2-4 above. The difference between the estimated composite score and the true composite score is evaluated as follows:

$$\mid \hat{S}(f_i) - S(f_i) \mid = \left| \sum_{j=1}^{\hat{m}} \hat{w}_j \hat{R}_j(f_i) - \sum_{j=1}^{m} w_j R_j(f_i) \right|.$$

Consider the case where $\hat{m} = m$ (i.e., the estimated number of segments matches the true number of segments):

$$\mid \hat{S}(f_i) - S(f_i) \mid = \left| \sum_{j=1}^{m} (\hat{w}_j \hat{R}_j(f_i) - w_j R_j(f_i)) \right| \leq \sum_{j=1}^{m} |\hat{w}_j \hat{R}_j(f_i) - w_j R_j(f_i)|.$$

For each segment j, the difference is further decomposed:

$$\begin{aligned} |\hat{w}_j \hat{R}_j(f_i) - w_j R_j(f_i)| &= |\hat{w}_j \hat{R}_j(f_i) - \hat{w}_j R_j(f_i) + \hat{w}_j R_j(f_i) - w_j R_j(f_i)| \\ &\leq \mid \hat{w}_j \mid \cdot \mid \hat{R}_j(f_i) - R_j(f_i) \mid + \mid R_j(f_i) \mid \cdot \mid \hat{w}_j - w_j \mid \end{aligned}$$

According to Theorems 3 and 4, the following holds when $n \to \infty$:

$$\mid \hat{w}_j - w_j \mid \xrightarrow{a.s.} 0,$$

and

$$\mid \hat{R}_j(f_i) - R_j(f_i) \mid \xrightarrow{p} 0.$$

Moreover, $\mid \hat{w}_j \mid$ and $\mid R_j(f_i) \mid$ are bounded by $\mid \hat{w}_j \mid \leq 1$ and $\mid R_j(f_i) \mid \leq n$, respectively. Therefore, according to the continuous mapping theorem:

$$|\hat{w}_j \mid \cdot |\hat{R}_j(f_i) - R_j(f_i)| \xrightarrow{p} 0 \, |R_j(f_i)| \cdot |\hat{w}_j - w_j| \xrightarrow{a.s.} 0.$$

This means that for each segment j, when condition $n \to \infty$ holds, $| \hat{w}_j \hat{R}_j(f_i) - w_j R_j(f_i) | \xrightarrow{p} 0$. Since the number of segments m is finite, by the convergence property of finite sums, we have

$$\sum_{j=1}^{m} \left| \hat{w}_j \hat{R}_j(f_i) - w_j R_j(f_i) \right| \xrightarrow{p} 0.$$

This indicates that when $\hat{m} = m$, $| \hat{S}(f_i) - S(f_i) | \xrightarrow{p} 0$.

Consider a more general case, specifically the possibility of $\hat{m} \neq m$. According to Theorem 2, if $n \to \infty$ holds, then $P(\hat{m} = m) \to 1$ follows. In other words, for any $\varepsilon > 0$, there exists an N such that when $n > N$ occurs, $P(\hat{m} \neq m) < \varepsilon$ is satisfied. The difference in the composite scores can be decomposed as:

$$\begin{aligned} P(| \hat{S}(f_i) - S(f_i) | > \delta) = P(| \hat{S}(f_i) - S(f_i) | > \delta \mid \hat{m} = m) P(\hat{m} = m) \\ + P(| \hat{S}(f_i) - S(f_i) | > \delta \mid \hat{m} \neq m) P(\hat{m} \neq m) \end{aligned}$$

It has been demonstrated above that when $\hat{m} = m$, $| \hat{S}(f_i) - S(f_i) | \xrightarrow{p} 0$ holds true, therefore, $P(| \hat{S}(f_i) - S(f_i) | > \delta \mid \hat{m} = m) \to 0$ is valid for any $\delta > 0$. Since $P(| \hat{S}(f_i) - S(f_i) | > \delta \mid \hat{m} \neq m)$ cannot exceed 1, it follows that

$$P(| \hat{S}(f_i) - S(f_i) | > \delta) \leq o(1) \cdot P(\hat{m} = m) + 1 \cdot P(\hat{m} \neq m) \to 0 + 0 = 0.$$

This demonstrates that when $n \to \infty$ occurs, $\hat{S}(f_i) \xrightarrow{p} S(f_i)$ holds, in other words, the estimated composite score converges to the true composite score in probability.

For any pair of functions (f_i, f_j), if $S(f_i) > S(f_j)$ holds, then there exists $\delta_{ij} > 0$ such that $S(f_i) - S(f_j) > \delta_{ij}$. Since $\hat{S}(f_i) \xrightarrow{p} S(f_i)$ and $\hat{S}(f_j) \xrightarrow{p} S(f_j)$ are true, it follows that when n is sufficiently large, we have

$$P(| \hat{S}(f_i) - S(f_i) | < \delta_{ij}/3) > 1 - \varepsilon/2 P(| \hat{S}(f_j) - S(f_j) | < \delta_{ij}/3) > 1 - \varepsilon/2.$$

According to the union bound of probability, the following can be derived:

$$P(| \hat{S}(f_i) - S(f_i) | < \delta_{ij}/3,$$

and

$$| \hat{S}(f_j) - S(f_j) | < \delta_{ij}/3) > 1 - \varepsilon.$$

In this case

$$\hat{S}(f_i) > S(f_i) - \delta_{ij}/3 > S(f_j) + 2\delta_{ij}/3 > S(f_j) + \delta_{ij}/3 > \hat{S}(f_j).$$

This means

$$P(\hat{S}(f_i) > \hat{S}(f_j) \mid S(f_i) > S(f_j)) > 1 - \varepsilon.$$

Since the number of functions n is finite, the number of function pairs in the ranking is also finite. By applying the above conclusion to all possible function pairs and using the union bound of probabilities, it can be shown that, when the sample size is sufficiently large, the probability that the estimated ranking matches the true ranking can be made arbitrarily close to 1. Therefore, the ranking result based on $\hat{S}(f_i)$ converges in probability to the true ranking result based on $S(f_i)$.

References

1. Wahba G. Spline models for observational data. Society for industrial and applied mathematics, 1990. doi:10.1016/0021-9045(91)90041-8

2. Stone CJ. Optimal global rates of convergence for nonparametric regression. The annals of statistics, 1982: 1040-1053. doi:10.1214/aos/1176345969

3. Craven P, Wahba G. Smoothing noisy data with spline functions: estimating the correct degree of smoothing by the method of generalized cross-validation. Numerische mathematik, 1978, 31(4): 377-403. https://doi.org/10.1007/BF01404567

4. Ester M, Kriegel HP, Sander J, et al. A density-based algorithm for discovering clusters in large spatial databases with noise. kdd. 1996, 96(34): 226-231. https://cdn.aaai.org/KDD/1996/KDD96-037.pdf?source=post_page

5. Sander J, Ester M, Kriegel HP, et al. Density-based clustering in spatial databases: The algorithm gdbscan and its applications. Data mining and knowledge discovery, 1998, 2: 169-194. https://doi.org/10.1023/A:1009745219419

6. López-Pintado S, Romo J. On the concept of depth for functional data. Journal of the American statistical Association, 2009, 104(486): 718-734. https://doi.org/10.1198/jasa.2009.0108

7. Arcones MA, Giné E. Limit theorems for U-processes. The Annals of Probability, 1993: 1494-1542. doi:10.1214/aop/1176989128
